# Using genomics to guide seed‐sourcing at the right taxonomical level for ecological restoration projects: The complex case of *Carex bigelowii* s.lat. in Norway

**DOI:** 10.1002/ece3.8350

**Published:** 2021-11-17

**Authors:** Kristine Bakke Westergaard, Magni Olsen Kyrkjeeide, Marie Kristine Brandrud

**Affiliations:** ^1^ Norwegian Institute for Nature Research Trondheim Norway

**Keywords:** alpine ecological restoration, *Carex bigelowii* s.lat., ddRAD‐seq, genetic structure, hybrids, seed‐sourcing, subspecies, taxonomic impediment

## Abstract

There is a growing demand for ecological restoration using suitable seeds following international standards or national legal demands for local seed‐sourcing. However, before selecting the appropriate geographic origin of seeds, it is vital to explore taxonomic complexity related to the focal taxa. We used ddRAD‐seq to screen genomic diversity within *Carex bigelowii* s.lat. focussing on Norway. This species complex is considered a candidate for seeding, but presents considerable morphological, ecological, and genetic variation. The genetic structure of 132 individuals of *C. bigelowii* s.lat., including *Carex nigra* as an outgroup, was explored using ordinations, clustering analyses, and a genetic barrier algorithm. Two highly divergent clusters were evident, supporting the recognition of two taxonomic units “*C. dacica*” and *C. bigelowii* “subsp. *bigelowii*”. Previously defined seed‐sourcing regions for *C. bigelowii* s.lat. did not consider the known taxonomic complexity, and therefore interpreted the overall genetic structure as seed‐sourcing regions, not taxa. We estimated genetic neighborhood sizes within each taxon to be 100–150 km and 300 km, respectively, indicating species‐specific delimitations of local seed‐sourcing regions. Frequent hybrids, local genetic distinctiveness, and suggested ecotypes add complexity to the discussed seed‐sourcing regions. Our results show how genomic screening of diversity and structure in a species complex can alleviate the taxonomic impediment, inform practical questions, and legal requirements related to seed‐sourcing, and together with traditional taxonomic work provide necessary information for a sound management of biodiversity.

## Graphical abstract

There is a growing demand for ecological restoration using suitable seeds following international standards or national legal demands for local seed sourcing. However, before selecting the appropriate geographic origin of seeds, it is vital to explore the taxonomic complexity related to the focal taxa. Genomic screening of diversity and structure of the complex case of *Carex bigelowii* s.lat. can alleviate the taxonomic impediment, inform practical questions and legal requirements related to seed sourcing, and together with traditional taxonomic work provide necessary information for a sound management of biodiversity.

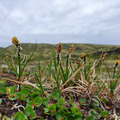

## 1 INTRODUCTION

Species and ecosystems are threatened by widespread, continued, and accelerating degradation and loss, leading to loss of ecosystem functioning and services (IPBES, 2019). Therefore, the UN General Assembly has stated that “there has never been a more urgent need to restore damaged ecosystems than now,” and declared the next decade 2021–2030 as the UN Decade on Ecosystem Restoration (https://www.decadeonrestoration.org/). Rebuilding ecosystems can be done by ecological restoration, which is the process of assisting the recovery of an ecosystem that has been degraded, damaged, or destroyed (Clewell et al., 2004). Importantly, any restoration should consider all variability found among living organisms, including diversity within species, between species, and of ecosystems (CBD, 1992).

A growing demand for ecological restoration increases the requirements for seeds (Gann et al., 2019). The use of native, regional, or local seeds to prevent maladaptation and in‐ or outbreeding depression when reintroducing plants is considered most successful in ecological restoration (Breed et al., 2018; Hagen et al., 2014). However, there has been a shift away from traditional local seed‐sourcing towards mixed and targeted approaches, broadening the genetic basis of a restored flora to maximize the long‐term success and resilience of restoration programs (Bucharova et al., 2019; Hoffmann et al., 2021). There are numerous proposed methods to evaluate the appropriate provenance and select a suitable seed‐sourcing strategy (summarized in e.g. Breed et al., 2013, 2018). Recently, genomic approaches have been applied to identify hierarchically ordered clusters of genetic diversity and differentiation in plant species, providing useful tools for delimiting seed‐sourcing regions (Breed et al., 2019; De Kort et al., 2014; Jørgensen et al., 2016; Williams et al., 2014).

An international standard for the use of native seeds in ecological restoration has just been published (Pedrini & Dixon, 2020), and seed transfer zones have been suggested for several countries (Bower et al., 2014; De Vitis et al., 2017), aiding restoration projects locally and nationally. In practical restoration of flora, there will be many biological unknowns when making decisions around seed‐sourcing, but they will nevertheless have to be made within the prevailing legal framework. In Norway, ecological restoration projects must follow the legal framework set by the Norwegian Nature Diversity Act of 2009 (https://lovdata.no/dokument/NL/lov/2009‐06‐19‐100?q=naturmangfoldloven) with associated regulations (https://lovdata.no/dokument/SF/forskrift/2015‐06‐19‐716), which instruct the use of plant material of local provenance without defining what local means. Based on AFLP markers, general seed transfer zones for commercial seed production of a set of common and widely distributed alpine plant species in Norway have been proposed (Jørgensen et al., 2016). However, before selecting the appropriate geographic origin of seeds for re‐introduction, it is vital to explore the taxonomic complexity related to the taxa in focus. By overlooking taxonomic complexity, one risks to misinterpret genetic structuring caused by the presence of taxonomic entities as seed‐sourcing regions.

Here, we focus on the diploid (2*n* = 68–70; Więcław et al., 2020) arctic‐alpine circumpolar sedge *C. bigelowii* Torr. Ex Schwein s.lat., a candidate for seeding in alpine restoration projects in Norway, for which a northern and a southern seed transfer zone have been identified (for *C. bigelowii* (sic); Jørgensen et al., 2016). However, *C. bigelowii* s.lat. is a taxonomically complicated species complex presenting considerable morphological, ecological, and genetic variation, and it is known to frequently hybridize within Scandinavia and elsewhere (Benítez‐Benítez et al., 2021; Brooker et al., 2001; Nakamatte & Lye, 2007, 2010; Schönswetter et al., 2008). Currently, two taxa are recognized in mainland Norway: *C. bigelowii* Torr. ex Schwein. subsp. *bigelowii* and *C. bigelowii* subsp. *dacica* (Heuff.) T. V. Egorova (Artsdatabanken, 2020a; Lid & Lid, 2005). The intraspecific division is described to be between plants growing in a wetter habitat (bogs) having a staminate spike with a peduncle, and blunt pistillate scales (*C. bigelowii* subsp. *bigelowii*), and plants growing in a dryer habitat (ridges) with a sessile staminate spike and more pointed pistillate scales (*C. bigelowii* subsp. *dacica*; Lid & Lid, 2005). *Carex bigelowii* subsp. *bigelowii* is described to be widely distributed in northern Norway and northeastern parts of southern Norway (Artsdatabanken, 2020b; Solstad et al., 2015; L. Galten, personal communication) while *C. bigelowii* subsp. *dacica* should be common throughout Norway. Thus, the current treatment of the two subspecies consider them morphologically and ecologically distinct, but broadly sympatric in Norway.

Unlike the described distribution of *C. bigelowii* s.lat. in Norway, three separate genetic studies including Scandinavian samples have all identified two distinct genetic clusters in the region; one in the north and one in the south. First, a northern Norwegian population of *C. bigelowii* subsp. *bigelowii* was found to fall into an American genetic cluster, and to be morphologically and genetically distinct from the European genetic cluster of *C. bigelowii* subsp. *rigida* (syn. subsp. *dacica*) containing a southern Norwegian population (Nakamatte & Lye, 2007). Likewise, a circumpolar phylogeographical study of *C. bigelowii* s.lat. found a genetic and geographical division of four Scandinavian populations into a northern group belonging to an amphi‐Atlantic cluster (*C. bigelowii* subsp. *bigelowii*), a southern group belonging to a European cluster (*C. bigelowii* subsp. *rigida*), but also an admixed population in between (Schönswetter et al., 2008). Again, the same genetic pattern of a northern and southern genetic group within *C. bigelowii* [*sic*] was found by Jørgensen et al. (2016). However, the latter two groups were not suggested to potentially represent the two subspecies, but instead hypothesized to represent different post‐glacial immigration routes into Norway, and further suggested as two seed transfer zones for commercial seed production. However, analyses of carpological features among species in *Carex* section *Phacocystis* found unique morphological types of achenes for *C. bigelowii* s. str. and *C. dacica*, each with diagnostic characters (Jiménez‐Mejías & Martinetto, 2013). Given the complex taxonomy of *C. bigelowii* s.lat., with taxa seemingly occurring in sympatry that are morphologically, ecologically, and genetically differentiated, we hypothesize that it cannot be treated as one taxon for delimiting intraspecific seed‐sourcing regions in Norway.

The recent introduction of genomic methods to ecological restoration biology is considered a promising tool for achieving restoration goals, for example by identifying proper seed transfer zones and monitoring the genomic outcome of restoration (Breed et al., 2019; Williams et al., 2014). However, there are still very few case studies showing the potential of using genomic tools in guiding ecological restoration (but see Carvalho et al., 2021; Wood et al., 2020). Here, we use double digest RAD sequencing (ddRAD‐seq) and genome‐wide SNP data to revisit the taxonomic uncertainty and discuss Norwegian seed‐sourcing regions for *C. bigelowii* s.lat.by screening the genomic diversity of Norwegian populations of *C. bigelowii* s.lat. with additional samples from North America and Europe. First, we investigate whether genetic structure found within *C. bigelowii* s.lat. in Norway corresponds to the described subspecies, whether they have overlapping geographical ranges, and explore admixture within and hybridization between genetic groups. We further investigate genetic and possibly geographic structure and diversity within the two recognized subspecies in Norway, and discuss the impact of our results on delimitation of seed‐sourcing regions.

## 2 MATERIALS AND METHODS

### 2.1 Sampling, library preparation, and sequencing

**FIGURE 1:**
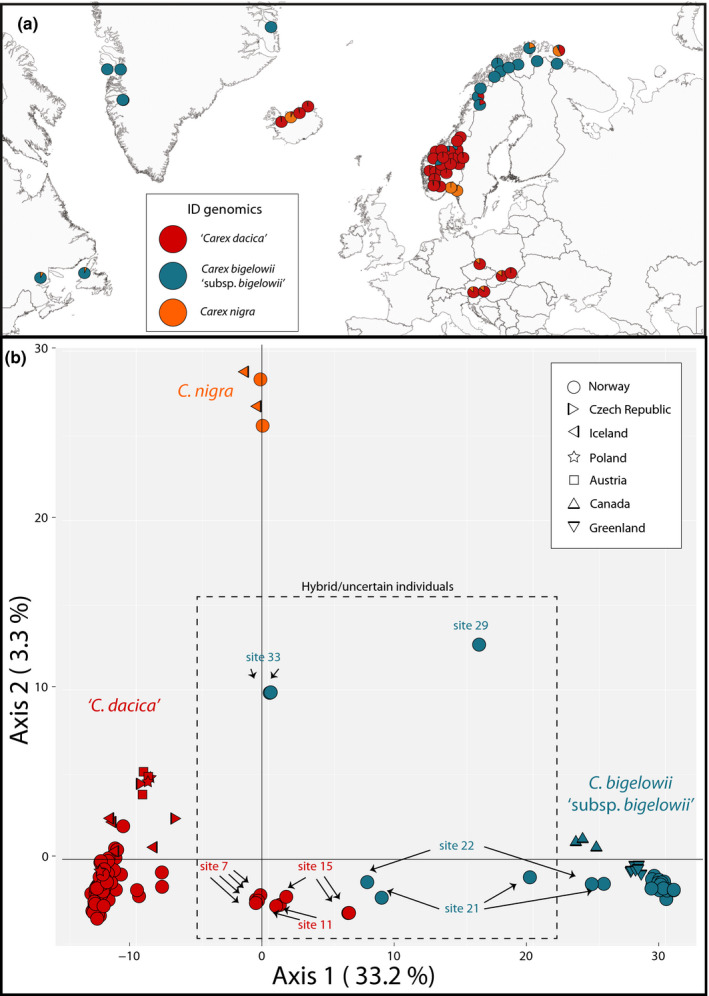
Main genetic structure of sampled *Carex bigelowii* Torr. Ex Schwein s.lat. and the outgroup *C. nigra* (L.) Reichard in the North Atlantic region based on: (a) STRUCTURE results for *K* = 3, where “*C. dacica*” is interpreted as the red cluster, *C. bigelowii* “subsp. *bigelowii*” as the blue cluster, and *C. nigra* as the orange cluster; and (b) Two‐dimensional PCoA plot (first and second axes) based on dataset A (15,095 SNPs) for all 132 individuals and one replicate, where the geographical origin of each individual is shown with different shapes, and interpreted hybrid or taxonomically uncertain individuals are identified to sites (see Table 1 for site information)

We collected 139 individuals of *C. bigelowii* s.lat. from 33 sites across its Norwegian distribution and 16 sites outside Norway (Figure 1a, Table 1). In addition, three individuals of *C. nigra* (L.) Reichard were included as outgroup. Because *C. bigelowii* s.lat. is known to reproduce clonally (e.g., Callaghan, 1976), the individuals were sampled several meters apart, as far from each other as possible given the spatial extent of the populations.

**TABLE 1:** Collection information for the 51 sampled sites of *Carex bigelowii* Torr ex. Schwein. s.lat. and *Carex nigra* sites: *n*—number of individuals collected/retained in analyses

Site ID	Site	Country	Lat	Long	*n*	ID morphology	ID genomics	Collector(s)^a^	Year	Voucher ID
1	Merakkhaugen	Norway	59.86	8.31	4/4	subsp. *dacica*	“*C. dacica*”	MOK, HEM, ME, RB	2017	pop11
2	Skiftessjøen	Norway	60.37	7.54	4/3	subsp. *dacica*	“*C. dacica*”	SLO	2017	pop25
3	Finse	Norway	60.58	7.57	4/4	cf. subsp. *bigelowii*	“*C. dacica*”	RR	2017	pop2
4	Børefjell	Norway	60.84	7.49	4/4	subsp. *dacica*	“*C. dacica*”	MJ	2017	pop14
5	Hemsedal	Norway	60.96	8.17	4/4	subsp. *dacica*	“*C. dacica*”	SLO	2017	pop23
6	Vikafjellet	Norway	60.93	6.41	4/4	subsp. *dacica*	“*C. dacica*”	SLO	2017	pop22
7	Memurudalen	Norway	61.50	8.53	4/4	cf. subsp. *dacica*	cf. “*C. dacica*”	SLO	2017	pop4
8	Ringebufjellet	Norway	61.56	10.30	4/4	subsp. *bigelowii*	“*C. dacica*”	MOK	2017	pop17
9	Sølen	Norway	61.85	11.58	4/3	subsp. *dacica*	“*C. dacica*”	ME, BGS	2017	pop9
10	Røvolltjønnan	Norway	62.33	12.04	4/4	subsp. *dacica*	“*C. dacica*”	ME	2017	pop6
11	Forollhogna	Norway	62.73	11.10	4/3	subsp. *dacica*	“*C. dacica*” (1) “subsp. *bigelowii*” × “*C. dacica*” (2)	SLO	2017	pop5
12	Hjerkinn	Norway	62.24	9.49	5/5	subsp. *dacica*	“*C. dacica*”	DH	2017	pop26
13	Snøhetta	Norway	62.34	9.32	4/4	subsp. *dacica*	“*C. dacica*”	HEM, AH	2017	pop13
14	Nordre Knutshø	Norway	62.32	9.67	4/4	subsp. *dacica*	“*C. dacica*”	HEM	2017	pop18
15	Leirtjønnkollen	Norway	62.46	9.75	7/7	subsp. *dacica* (1) subsp. *bigelowii* (6)	“*C. dacica*” (4) “subsp. *bigelowii*” × “*C. dacica*” (3)	HEM	2017	pop15
16	Stryn	Norway	62.07	7.10	4/4	subsp. *dacica*	“*C. dacica*”	RB	2017	pop8
17	Trollstigen	Norway	62.44	7.71	5/5	subsp. *dacica* (4) cf. subsp. *bigelowii* (1)	“*C. dacica*”	NHT	2017	pop21
18	Grødalen	Norway	62.52	8.93	4/4	subsp. *dacica*	“*C. dacica*”	SLO	2017	pop3
19	Hårskallen	Norway	63.65	11.51	4/4	subsp. *dacica*	“*C. dacica*”	MOK	2017	pop16
20	Ogndalen	Norway	63.99	12.13	1/1	subsp. *Bigelowii* × *C. nigra*	“*C. dacica*”	NHT	2017	pop20
21	Junkerdalen	Norway	66.98	15.84	4/4	subsp. *bigelowii*	“subsp. *bigelowii*” (2–3) “subsp. *bigelowii*” × “*C. dacica*” (1–2)	DH	2017	pop19
22	Fauske	Norway	67.41	15.29	2/2	subsp. *bigelowii* × *C. nigra*	“subsp. *bigelowii*” (1) “subsp. *bigelowii*” × “*C. dacica*” (1)	BGØ	2017	pop24
23	Hamarøy	Norway	67.87	15.84	2/1	subsp. *bigelowii*	“subsp. *bigelowii*”	RE, TMP	2013	O‐DP−61804
24	Målselv	Norway	68.88	19.63	3/3	subsp. *dacica*	“subsp. *bigelowii*”	TMP, RE	2012	O‐DP−55584/−86/−88
25	Norddalen	Norway	69.23	20.09	3/3	subsp. *bigelowii*	“subsp. *bigelowii*”	MOK, AO	2017	pop7
26	Kåfjord	Norway	69.37	21.10	1/1	subsp. *dacica*	“subsp. *bigelowii*”	TMP, RE	2012	O‐DP−55498
27	Tromsø	Norway	69.63	18.99	6/4	subsp. *dacica* (2) cf. subsp. *bigelowii* (2)	“subsp. *bigelowii*”	MOK, AO	2017	pop1
28	Kvænangen	Norway	69.90	21.60	1/1	subsp. *bigelowii*	“subsp. *bigelowii*”	TMP	2012	O‐DP−55636
29	Kvalsund	Norway	70.26	24.09	2/2	subsp. *bigelowii*	“subsp. *bigelowii*” “subsp. *bigelowii*” × *C. nigra*	TMP, RE, CSB	2012	O‐DP−55813/O‐DP−55625
30	Porsanger	Norway	70.36	25.97	1/1	subsp. *dacica*	“subsp. *bigelowii*”	TMP, RE, CSB	2012	O‐DP−55842
31	Sør‐Varanger	Norway	69.97	29.60	3/3	subsp. *dacica*	“subsp. *bigelowii*”	TMP, RE, CSB	2012	O‐DP−55890/−89/−91
32	Vardø	Norway	70.42	30.71	1/0	subsp. *dacica*	n/a	TMP, RE, CSB	2012	O‐DP−55937
33	Båtsfjord	Norway	70.51	30.44	2/2	subsp. *bigelowii*	cf. “subsp. *bigelowii*” × “*C. dacica*” × *C. nigra*	TMP, RE, CSB	2012	O‐DP−55423/−25
34	Kangerlussuaq, Ravneklippen	Greenland	67.01	−50.67	2/2	*C. bigelowii*	“subsp. *bigelowii*”	RE, TMP	2013	O‐DP−62164/−66
35	Kangerlussuaq, Store Saltsø	Greenland	66.99	−50.60	2/2	*C. bigelowii*	“subsp. *bigelowii*”	RE, TMP	2013	O‐DP−62183/−85
36	Ilulissat	Greenland	69.23	−51.08	2/1	*C. bigelowii*	“subsp. *bigelowii*”	RE, TMP	2013	O‐DP−62191/−94
37	Qeqertarsuaq	Greenland	69.25	−53.54	2/2	*C. bigelowii*	“subsp. *bigelowii*”	RE, TMP	2013	O‐DP−62211/−13
38	Antarctichavn	Greenland	71.99	23.11	1/1	*C. bigelowii*	“subsp. *bigelowii*”	CB, AKB	2004	O‐DP−26149
39	Melrakkasletta	Iceland	66.49	−16.25	1/1	*C. bigelowii*	“*C. dacica*”	CSB, TMP	2009	O‐DP−62025
40	Gasir	Iceland	65.78	−18.17	2/2	*C. bigelowii*	“*C. dacica*”	CSB, TMP	2010	O‐DP−62094/−96
41	Hrutajördur	Iceland	65.26	−21.15	1/1	*C. bigelowii*	“*C. dacica*”	CSB, TMP	2013	O‐DP−62129
42	Quebec	Canada	49.00	65.94	2/2	*C. bigelowii*	“subsp. *bigelowii*”	PS, AT	2004	O‐DP−4283/−90
43	Newfoundland	Canada	49.60	57.79	1/1	*C. bigelowii*	“subsp. *bigelowii*”	IGA, AKB	2004	O‐DP−4293
44	Schmelz	Austria	47.09	14.55	2/1	*C. bigelowii*	“*C. dacica*”	AT, PS	2003	O‐DP−26420/−28
45	Ladinger Spitz	Austria	46.85	14.65	2/2	*C. bigelowii*	“*C. dacica*”	AT, PS	2003	O‐DP−26356/−58
46	Mt. Zadni Ornak	Poland	49.21	19.84	2/2	*C. bigelowii*	“*C. dacica*”	PS, MR	2004	O‐DP−4258/−59
47	Tatra Mountains	Poland	49.20	19.84	1/1	*C. bigelowii*	“*C. dacica*”	PS, MR	2004	O‐DP−4267
48	Nordböhmen, Riesengebirge	Czech Republic	15.69	50.72	2/2	*C. bigelowii*	“*C. dacica*”	LSE	2003	O‐DP−26307/−08
49	Tønsberg	Norway	59.28	10.37	2/1	*C. nigra*	*C. nigra*	CSB, RE, TMP	2013	O‐DP−61711/−12
50	Halden	Norway	58.98	11.48	1/1	*C. nigra*	*C. nigra*	CSB, RE, TMP	2013	O‐DP−61699
51	Skipalon	Iceland	65.79	−18.20	2/2	*C. bigelowii*	*C. nigra*	CSB, TMP	2013	O‐DP−62091/−92

Note

ID morphology—morphological identification of Norwegian specimens of *C. bigelowii* s.lat. (subsp. *bigelowii* and subsp. *dacica*) and potential hybrids according to differential characters (Lid & Lid, 2005), foreign material identified as *C. bigelowii* [*sic*] by collectors (number of individuals in parentheses). ID genomics—suggested genomic clusters of *C. bigelowii* s.lat. based on our ddRAD‐seq results (number of individuals in parentheses). Voucher ID – “pop” material stored at Norwegian Institute for Nature Research, “O‐DP” DNA bank accession nos. at NHMO DNA Bank at the Natural History Museum, University of Oslo.

^a^ Collectors: AH Annika Hofgaard, AKB Anne Krag Brysting, AO Anders Often, AT Andreas Tribsch, BGS Bård Gunnar Stokke, BGØ Bernt‐Gunnar Østerkløft, CB Christian Brochmann, CSB Charlotte Sletten Bjorå, DH Dagmar Hagen, HEM Heidi Elin Myklebost, IGA Inger Greve Alsos, LSE Luise Schratt‐Ehrendorfer, ME Marianne Evju, MJ Mari Jokerud, MOK Magni Olsen Kyrkjeeide, MR Michal Ronikier, NHT Neri Horntvedt Thorsen, PS Peter Schönswetter, RB Rakel Blaalid, RE Reidar Elven, RR Ruben Roos, SLO Siri Lie Olsen, TMP Tiril Myhre Pedersen.

**FIGURE 2:**
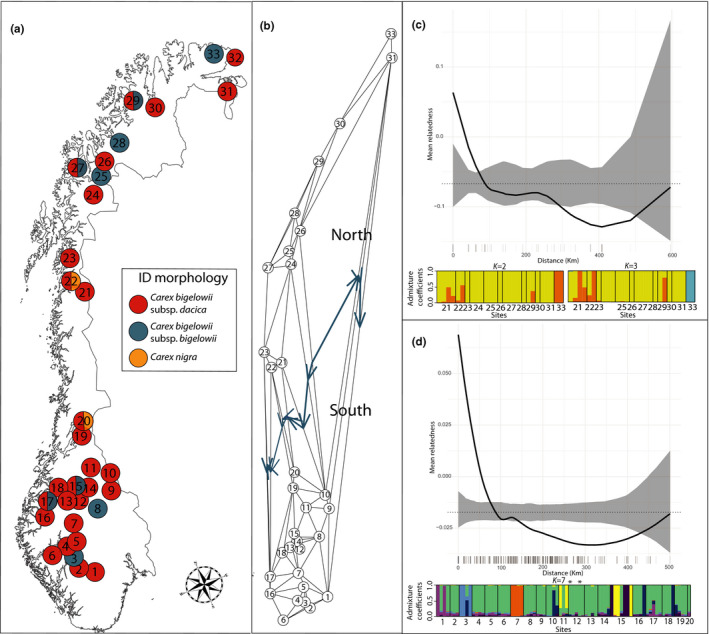
(a) Morphologically identified Norwegian samples of *Carex bigelowii* Torr. Ex Schwein s.lat. and the outgroup *C. nigra* (L.) Reichard according to the differential characters given in the Norwegian Flora (Lid & Lid, 2005). (b) genetic barriers within *C. bigelowii* s. lat based on dataset B (5,134 SNPs) in Norway detected by the Monmonier maximum‐difference algorithm (visualized as blue arrows), (c) fine‐scale spatial genetic structure (17 individuals, excluding hybrids and uncertain individuals) and genetically homogeneous groups detected by sparse Nonnegative Matrix Factorization (sNMF; *K* = 2 and *K* = 3) within the northern Norwegian cluster *C. bigelowii* ‘subsp. *bigelowii*’ using dataset B, d) fine‐scale spatial genetic structure (64 individuals, excluding hybrids and uncertain individuals) and genetically homogeneous groups detected by sNMF (*K* = 7) within the southern Norwegian cluster ‘*C. dacica*’ (the replicated individual from 12 Hjerkinn is marked with an asterisk) using dataset B. For the estimations of genetic neighborhood sizes, the black dotted lines show the null expectation, the shaded areas show the 95% confidence around the null expectation, and the black solid lines show the LOESS fit to the observed relatedness

According to the differential morphological characters given in the Norwegian Flora (Lid & Lid, 2005), each Norwegian individual was tentatively determined to *C. bigelowii* subsp. *bigelowii* (eight sites), *C. bigelowii* subsp. *dacica* (20 sites), a hybrid between the two subspecies (three sites), or even a hybrid with *C. nigra* (two sites; Figure 2a, Table 1). All specimens collected outside of Norway were identified as *C. bigelowii* [*sic*] by the respective collectors.

Genomic DNA from silica‐dried leaf material was extracted using NucleoSpin Plant II extraction kit (Macherey–Nagel) following the manufacturer's protocol, and quantified on a Qubit 2.0 using the HS Assay kit (Thermo Fisher Scientific), before ddRAD‐seq libraries were prepared following the protocols described in Westergaard et al. (2019). One sample was replicated from DNA extraction through all analyses. The libraries were sequenced in four lanes of 81‐bp paired‐end reads on an Illumina NextSeq 500 at the Genomic Core Facility (GCF), Norwegian University of Technology and Science, Norway.

### 2.2 De novo assembly of loci, variant calling, and filtering

The six part‐libraries were demultiplexed and quality checked separately using the *process_radtags* component of STACKS v. 2.3e, before de novo assembly of a catalogue of loci, and calling of SNPs and genotypes with the component *denovo_map* (Catchen et al., 2011, 2013). The settings applied for the de novo assembly were chosen following the recommendations of parameter settings by Paris et al. (2017). In brief, our study is not phylogenetic, our data was clean (i.e., 98.68% perfect index and 97.50% retained reads), and samples were collected at different sites (“populations”); therefore, [‐*m* 3] and [‐*m* 5], [‐M 2] and [‐M 3], and n = M are recommended to be tested. *Splitstrees* (Huson & Bryant, 2005) were drawn for [−m 3, −M 2, −n 2] and [−m 5, −M 3, −n 3] to check for topological difference (none found, but higher number of SNPs and loci for [−m 3 −M 2 −n 2], thus these parameter settings were used for the subsequent analyses).

Altogether, 10 individuals collected from 10 different sites were excluded from the final analyses as they had more than 50% missing data (Table 1). Two separate datasets were prepared for the downstream analyses of genetic structure and diversity: dataset A allowing all SNPs for ordinations (15,095 SNPs, including linked markers from the same locus), and dataset B allowing only one SNP per locus (5,134 SNPs, approximating unlinked markers) to meet the assumption of independent markers for all other analyses. The datasets were generated with the *populations* component of STACKS, allowing 10% missing data across loci, 80% maximum observed heterozygosity, and a minimum allele frequency (MAF) that would require an allele to be present in more than one individual, that is, min‐maf 0.01. For a more detailed description of the SNP calling and filtering, including a MAF value sensitivity analysis showing the effect on downstream analyses, see Appendix A, Table S1 and Figures S1–S4. Lastly, *F*_st_ values were calculated for all Norwegian sites using *populations*, excluding single individual sites for mean *F*_st_ values.

### 2.3 Genetic structure and diversity

The genetic structuring of the data was first explored for all individuals using dataset A in a principal coordinates analysis (PCoA) with Euclidean distance using the dartR package (Gruber et al., 2018) in RSTUDIO v. 1.0.44 (R Team, 2020). Next, we used clustering analyses to investigate the genetic separation of the morphologically defined taxa, that is, *C. bigelowii* subsp. *bigelowii*, *C. bigelowii* subsp. *dacica*, and the outgroup *C. nigra*, and potential indications of admixture between them (sensu Meirmans, 2015; van Hengstum et al., 2012). For this purpose, we first ran a Discriminant Analysis of Principal Components (DAPC) for all individuals using dataset A and the R package adegenet v.2.1.1 (Jombart, 2008; Jombart & Ahmed, 2011). Clusters were generated using the *find*.*clusters()* function (max no. 51 = all sites), 140 PCs were kept, and *K* = 3 was chosen based on the “elbow” of the Bayesian Information Criterion (BIC) curve. The *dapc* function was run on the three clusters identified, keeping 100 PCs and two discriminant analysis functions. Second, we ran STRUCTURE v.2.3.4 (Falush et al., 2007; Pritchard et al., 2000), using dataset B for *K* = 1–4 and including all individuals, with an a priori expectation of *K* = 3, while allowing for a potential fourth genetic cluster. We applied an admixture model, default settings with 10 replicate runs, each with 1,000,000 iterations and a burn‐in of 100,000. STRUCTURE HARVESTER web v. 0.6.94 (Earl & vonHoldt, 2012) was used to summarize the runs. To implement geographical information in our assessment of overall genetic structuring of the data, the TESS3 algorithm (Caye et al., 2016) was applied for 10 runs per *K* (1–10) using dataset B and the TESS3r R‐package (Caye et al., 2018). The “elbow” of the cross‐validation score curve was evaluated to decide which *K* best describe the data, and *K* = 3 was visualized with a bar plot and interpolated on a geographic map. The dataset was scanned for outlier loci using an Fst approach implemented in the program (Francois et al., 2016; Martins et al., 2016).

To assess how many SNPs support each interpreted taxonomic entity (i.e., taxon‐specific SNPs), the “gl.filter.pa” function was applied with the dartR package using dataset A to calculate fixed (homozygote AA in one group and homozygote aa in the other group) and private alleles (heterozygote Aa in one group and homozygote aa) separating *C. bigelowii* “subsp. *bigelowii”* from “*C. dacica*,” *C. bigelowii* “subsp. *bigelowii*” from *C. nigra*, and “*C. dacica*” from *C. nigra*. Sites with interpreted hybrids or uncertain taxonomic affiliation (7, 11, 15, 21, 22, 29, 33; see Table 1 “ID genomics,” Figure 1) were grouped separately for these calculations.

To look for potential genetic barriers within *C. bigelowii* s. lat in Norway, the Monmonier maximum‐difference algorithm (Monmonier, 1973) was used to detect boundaries within dataset B (excluding *C. nigra*) using the package adegenet in R. Two main genetic clusters were recognized within *C. bigelowii* s. lat. (see Results: geographically structured to southern and northern Norway; Figure 2), and the data was further analyzed separately to explore seed‐sourcing regions within each cluster.

The presence of genetic groups within each of the southern and northern Norwegian genetic cluster of *C. bigelowii* s.lat. was explored with sparse Nonnegative Matrix Factorization (sNMF; Frichot et al., 2014) using the LEA v.2.4.0 package in R (Frichot & François, 2015) on dataset B, testing *K* = 1–15 for southern Norway and *K* = 1–10 for northern Norway. Both analyses were run with 10 replicates for each *K*, and otherwise default settings. To identify optimal *K*s, the “elbow” entropy values (where entropy stabilizes) were considered. The genetic neighborhood size and spatial autocorrelation of the fine‐scale genetic structure within the two main Norwegian genetic clusters (excluding hybrid/uncertain individuals; Table 1, Figure 1) were assessed by first calculating genetic relatedness between pairs of individuals according to Yang et al. (2010), and then fitting pairwise genetic relatedness and geographical distances with local polynomial fitting (LOESS), applying the Lplot script (https://github.com/rojaff/Lplot) and same settings as Carvalho et al. (2019).

All our results showed Site 33 Båtsfjord as being highly deviant. To see if this site is better explained as a distinct cluster or an admixture of two or three clusters, we performed a test with sNMF allowing admixture, similar to what was performed using STRUCTURE by van Hengstum et al. (2012). To avoid bias occurring due to unbalanced sampling (Meirmans, 2019), the test was run with a selection of individuals from the three clusters recognized as our main taxonomic entities (four *C. nigra*, eight “*C. dacica*,” nine *C. bigelowii* “subsp. *bigelowii*”*)*, and the two individuals from site 33 Båtsfjord with unknown origin/taxonomy for *K* = 3 and *K* = 4, each with 10 replicates. The “elbow” entropy values were considered to evaluate the genetic composition of site 33 Båtsfjord.

## 3 RESULTS

### 3.1 Genetic clusters support two taxa of *C. bigelowii* s.lat. in mainland Norway

The PCoA of dataset A showed that individuals from southern Norway, Iceland, and Central Europe (hereafter referred to as “*C. dacica*”) were highly separated from individuals in northern Norway, Canada, and Greenland (hereafter referred to as *C. bigelowii* “subsp. *bigelowii*”) at the first axis (33.2%; Figure 1). The second axis separated the outgroup *C. nigra* from all *C. bigelowii* s.lat. (3.3%), however, intermediate individuals were found in between all three main clusters. DAPC analyses indicated three clusters: one for “*C. dacica*,” one for *C. bigelowii* “subsp. *bigelowii*,” and one comprising *C. nigra* and the intermediate individuals apparent in the PCoA (Figures S5 and S6). Three main genetic clusters were also evident from the STRUCTURE analyses (Figure 1), and calculations of Pr[X|K] supported *K* = 3 (Figures S7 and S8). When *K* = 3, *C. nigra* formed a distinct cluster with a uniform genetic structure (also found by Jiménez‐Mejías et al., 2012), while *C. bigelowii* s. lat. was divided into two main clusters of individuals corresponding to the PCoA clusters, also recognized by TESS3 (Figures S9, S10, S11). No outlier loci were identified with a Benjamini‐Hochberg corrected False Discovery Rate set for the p value of 0.0001 (Figures S12, S13). The calculations of taxon‐specific SNPs found that most of the SNPs were specific for each interpreted taxon (i.e., *C. bigelowii* “subsp. *bigelowii*”, “*C. dacica*,” and *C. nigra*; Table S2), although there was some overlap. When *K* = 4, site 33 Båtsfjord in northern Norway formed a separate STRUCTURE cluster (data not shown). The two individuals were morphologically determined as *C. bigelowii* subsp. *bigelowii*, but seemed genetically admixed involving all three taxonomic entities when *K* = 3 (Figures 1, S8, and S10). Furthermore, this site formed a fourth cluster in a separate sNMF test (Figure S14) and separated from all other individuals along the third axis of the PCoA (Figure S15).

Individuals from the Icelandic site 51 Skipalon were morphologically identified as *C. bigelowii* [*sic*], but grouped closely with *C. nigra* in all analyses, and were thus considered misidentified. At site 29 Kvalsund, one individual was morphologically determined to *C. bigelowii* subsp. *bigelowii*, but genetically admixed between *C. bigelowii* “subsp. *bigelowii*” × *C. nigra* (Table 1; Figures 1, S8, S10). At site 20 Ogndalen, the single individual was morphologically determined as *C. nigra* × *C. bigelowii* subsp. *bigelowii*, but clustered genetically with “*C. dacica*.” The replicated individual from site 12 Hjerkinn showed highly reproducible results (Figure 2).

In general, genetic distance was high between sites in northern and southern Norway (mean *F*_ST_ = 0.33). Both the PCoA (Figure 1) and the *F*_ST_ values (Figure S16) showed low genetic distances between individuals from southern Norway (mean *F*_ST_ = 0.13); hence, it was recognized as a relatively uniform group. Apart from the genetically distant site 33 Båtsfjord, the sites from northern Norway were also more or less uniform (mean *F*_ST_ = 0.18, or mean *F*_ST_ = 0.29 with Båtsfjord included).

Even though a genetic barrier was detected in Norway between “*C. dacica*” and *C. bigelowii* “subsp. *bigelowii*” by the Monmonier maximum‐difference algorithm (Figure 2), swarms of hybrid individuals with intermediate genetic and morphological characteristics were also observed (Figure 1; Table 1). These individuals were not restricted to a geographic meeting zone in between the mainly northern *C. bigelowii* “subsp. *bigelowii*” and the southern “*C. dacica*.” At the southern site 15 Leirtjønnkollen, six out of seven individuals were initially morphologically determined to be the northern *C. bigelowii* subsp. *bigelowii*, of which three formed a separate cluster, while three were interpreted as hybrids between “*C. dacica*” and *C. bigelowii* “subsp. *bigelowii*” based on their admixed STRUCTURE results (Figure 1, S8), position in the hybrid swarm in the PCoA (Figure 1), and they also formed a separate cluster in the sNMF analyses of the southern cluster (Figure 2). Following the same hybrid pattern are site 7 Memurudalen and two out of three individuals from site 11 Forollhogna, and also the northern sites 21 Junkerdalen (one or two out of four individuals) and 22 Fauske (one out of two individuals).

### 3.2 Genetic structure within *C. bigelowii* “subsp. *bigelowii*” and “*C. dacica*” in Norway

In northern Norway, the cross‐entropy values for sNMF clusters plateaued between *K* = 2 and 3 (Figure S17), where site 33 Båtsfjord formed the third cluster (Figure 2). A major cluster contained most individuals, while a minor cluster contained single and admixed individuals from sites 22 Fauske, 21 Junkerdalen, and 30 Porsanger. Although the northern Norwegian genetic cluster appeared relatively uniform, we detected low levels of spatial autocorrelation with a large genetic neighborhood size (ca. 300 km, Figure 2). In southern Norway, the cross‐entropy values for sNMF clusters plateaued between *K* = 7 and 10 (Figure S17, *K* = 7 visualized in Figure 2), with no difference in resolution found within that range of *K*‐values. A major cluster prevailed in most sites and individuals, while minor gene pools prevailed in certain individuals, or appeared as admixed within other individuals (Figure 2). Site 7 Memurudalen formed a completely separate cluster. Three out of four individuals at site 3 Finse consistently formed a separate cluster, while the seven individuals at site 15 Leirtjønnkollen belonged to three different clusters: one interpreted as “*C. dacica*,” three to a minor gene pool of their own, and three interpreted as the hybrid *C. bigelowii* “subsp. *bigelowii*” × “*C. dacica*” also found in two individuals from site 11 Forollhogna. Low levels of genetic structuring were found in southern Norway, and the fine‐scale genetic analysis found that the southern individuals were spatially autocorrelated with an estimated genetic neighborhood size of ca. 100–150 km (Figure 2).

## 4 DISCUSSION

Knowledge about the speciation continuum is of particular importance when restoring degraded nature; otherwise, there is a risk of introducing foreign species and genotypes, biodiversity loss, and eventually, altered or even deteriorated ecosystem services (e.g., Alexander et al., 2016; Hagen et al., 2014). Our results illustrate how overlooked taxonomical complexity within a species complex, in this case one recommended for commercial seed production, affects the discussions and delimitation of seed‐sourcing regions. Inaccurate species identification pertaining to ecological information is a fundamental impediment to reducing biodiversity loss and restoring functional ecosystems (CBD, 2008). Although species often are used as units for management, this is complicated for species complexes where taxa are difficult to distinguish and hybridization occurs.

### 4.1 Overlooked taxonomical complexity is challenging

Our genomic screening focusing on Norway revealed that there are two unique and highly divergent evolutionary clusters within the common and widespread *C. bigelowii* s.lat., with a genomic distance even surpassing levels of species divergence found between *C. bigelowii* s.lat. and the outgroup *C. nigra*. Our results agree with the recent finding of two well‐defined, distantly related phylogenetic groups recognized as *C. bigelowii* s.s. and *C. dacica*, clearly supporting the recognition of *C. dacica* as a separate species (Benítez‐Benítez et al., 2021). Most authors consider *C. bigelowii* subsp. *bigelowii* and *C. dacica* to be closely related (e.g., Chater, 1980; Egorova, 1999; Nakamatte & Lye, 2007). However, the two clusters do not correspond to *C. bigelowii* subsp. *bigelowii* and subsp. *dacica* as described in the Norwegian Flora, supposedly occurring in broad sympatry (Lid & Lid, 2005). In contrast to reports of both subspecies being widely distributed, but ecologically differentiated (wet vs. dry habitats), we found them to be mainly geographically clustered in northern and southern groups. The northern group of *C. bigelowii* “subsp. *bigelowii”* clustered with individuals from Greenland and Canada, while the southern group of “*C. dacica*” clustered with individuals from Iceland and central Europe, following an overall pattern reported for European populations by Schönswetter et al. (2008) and Alsos et al. (2015).

Proper identification of *Carex* taxa often relies on few and subtle morphological characters, and demands a good overview of phenotypic plasticity within taxa and their ecology, often complicated by the frequent occurrence of partly or fully fertile hybrids (e.g., Cayouette & Catling, 1992; Nakamatte & Lye, 2010). Frequent hybridization within *Carex* section *Phacocystis* (including *C. bigelowii* s.lat. and *C. nigra*) further blurs taxon limits in this section (Duman & Kryszczuk, 1958; Lepage, 1956; Pedersen, 2017; Polunin, 1940; Raymond, 1952). Our results revealed a swarm of hybrids between *C. bigelowii* “subsp. *bigelowii”* and “*C. dacica*,” and also with *C. nigra*, but hybrids were neither easily recognized morphologically (Lid & Lid, 2005; Table 1) nor restricted to a geographically delimited hybrid zone. Admixed individuals were found distributed in large parts of the sampled area from sites 21 Junkerdalen and 22 Fauske in the north, to sites 11 Forollhogna and 15 Leirtjønnkollen in the south. The extent of hybridization and the origin of hybrids, including other closely related species in section *Phacocystis*, need further exploration.

*Carex bigelowii* [*sic*] is reported to mainly reproduce by runners (Callaghan, 1976), but seedling recruitment has been observed in disturbed sites like new road verges and fresh river gravel bars both in mountains and in the Arctic (Jonsson et al., 1996; Schönswetter et al., 2008; Stenström, 1999). The main clonal reproduction may explain the reduced gene flow between the interfertile *C. bigelowii* “subsp. *bigelowii*” and “*C. dacica*” in Norway, maintaining a general genetic structure with a southern and a northern group. Although the seeds of *C. bigelowii* s.lat. have no obvious morphological adaptations to long‐distance dispersal, previous multilocus assignment tests have revealed postglacial, trans‐Atlantic dispersal of both subspecies (Alsos et al., 2015), indicating a long‐distance dispersal potential. The site 33 Båtsfjord is located in the northernmost part of Norway, far away from any known “*C. dacica*” population, but our results indicate that the individuals may be admixed of *C. bigelowii* “subsp. *bigelowii*,” “*C. dacica*” and *C. nigra*. This site is genetically distant from near‐by site 31 Sør‐Varanger (Figure 2), and Jørgensen et al. (2016) also found another nearby population to be genetically “completely separated from the remaining.” They hypothesized a polemochoric (i.e., war dispersed) origin, as many species were introduced to this region during World War II (Alm et al., 2009).

Few case studies report on the consequences of undeliberate seeding of maladapted cryptic taxa or genetic lineages during ecological restoration, which might reflect that unsuccessful seeding is not reported, or the cause of failure is not identified (Hobbs, 2009). However, different negative effects of using commercially produced seed mixtures have been observed at restored sites (e.g., Aavik et al., 2012; Hagen et al., 2014), and several ecological studies report on intraspecific differences without reflecting on taxonomic uncertainties. For example, *C*. *bigelowii* [*sic*] is often considered a model species for long‐lived, arctic‐alpine clonal species because of the extensive data that exist on its population dynamics and demographic responses to a range of environmental factors (e.g., Carlsson & Callaghan, 1994; Jonsson et al., 1996; Little et al., 2015). However, by neither exploring nor acknowledging taxonomic complexity, studies on *C. bigelowii* [*sic*] including sites from different continents may have been conducted on different taxa reported by the same name, which in retrospect may cause confusion and even question the validity of interpreted results. For instance, Stenström (1999) found differences in the sexual reproductive ecology of *C. bigelowii* [*sic*] in northern Sweden and Iceland, and discussed how climatic or genetic differences affecting pollination and pollen viability might explain this, while Little et al. (2015) also treated *C. bigelowii* [*sic*] as a species, and reported highly species‐specific responses to global changes. Our results fall in line with a previous study (Schönswetter et al., 2008), supporting the recognition of Icelandic populations as *C. dacica*, while northern Swedish populations are most probably *C. bigelowii* subsp. *bigelowii*, or potential hybrids.

### 4.2 Implications for defining seed‐sourcing regions

Species delimitation is a highly contested area, and there is disagreement about almost every aspect of how to define and use the “species” category (see e.g., Padial & De la Riva, 2021; Raposo et al., 2020; Stanton et al., 2019). A thorough taxonomic and nomenclatural revision of the entire *C. bigelowii* species complex is clearly needed, but our results can be used to delineate seed‐sourcing regions relevant for ecological restoration in Norway under the assumption that genome‐wide likeness is a useful criterion for this purpose. According to the Norwegian Nature Diversity Act, the management goal for species is to ensure viable populations in their natural distribution areas while also attending to their genetic diversity (§4); to avoid damage to biodiversity (§6); and apply a precautionary principle when there is little knowledge on the effects of a measure. Our genomic results show that *C. bigelowii* “subsp. *bigelowii*” and “*C. dacica*” are highly separated, supporting a growing body of literature on the recognition of the two taxonomic units belonging to different species. Therefore, seed‐sourcing regions must be discussed separately within each taxon. The essential difference between our northern and southern Norwegian clusters, and the geographically similar population clusters reported by Jørgensen et al. (2016), is in the taxonomical hierarchy and subsequent considerations of intraspecific genetic diversity relevant for delimitations of seed‐sourcing regions. This affects restoration guidance, as seed‐sourcing regions will no longer be based on an intraspecific division between two population groups, or between supposedly broadly sympatric subspecies growing in different habitats throughout Norway.

Seed‐sourcing regions, seed (transfer) zones, or local provenance areas refer to regions that have similar environments in which natural genetic exchange occurs so that transfer of plant material within the zones should have little or no negative impact (Hufford & Mazer, 2003; Vander Mijnsbrugge et al., 2010). Further, a seed batch is considered appropriate for ecological restoration purposes when its genetic diversity represents its population of origin, and is used on a restoration site of suitable ecological conditions (Erickson & Halford, 2020; Pedrini & Dixon, 2020). Based on the expectation that populations are locally adapted, the idea that the use of so‐called local seeds always is the best seed‐sourcing strategy is, however, a subject for debate. Other strategies include composite provenancing (regional), admixture provenancing, climate‐adjusted, and predictive provenancing to enhance a species’ adaptive potential (Breed et al., 2013; Bucharova et al., 2019; Havens et al., 2015; Prober et al., 2015). For a widespread species like *Daucus carota* (wild carrot), non‐local seeds will work just as well as local seeds for restoration (Reiker et al., 2015), while seed‐sourcing regions for the endemic *Eucalyptus melliodora* in Australia have been defined to range 500 km (Supple et al., 2018), the distances separating sites within *C. bigelowii* “subsp. *bigelowii*” and “*C. dacica*.” Supple et al. (2018) further suggested that seeds could be sourced more broadly from favorable sites with higher seed quality, increasing genetic diversity without mixing divergent linages. Also, for *Eucalyptus cuprea*, mixed material was recommended to be used for restoration (Sampson & Byrne, 2016).

Estimates of genetic neighborhood size may, together with our assessments of genetic structure and diversity, inform the choice of donor sites and guide sampling distance for seeds of each of *C. bigelowii* “subsp. *bigelowii*” and *C. dacica* in Norway. The spatial autocorrelation in genetic relatedness varied between the two taxa. Low levels of spatial autocorrelation were found in the northern *C. bigelowii* “subsp. *bigelowii*,” and a large genetic neighborhood size of up to 300 km was estimated. Apart from interpreted hybrid populations, the sNMF analyses found little genetic structure within *C. bigelowii* “subsp. *bigelowii*.” Thus, seed sources for *C. bigelowii* “subsp. *bigelowii*” in northern Norway located within a 300 km radius can be considered “local” (cf. Norwegian Nature Diversity Act) for ecological restoration purposes. For the southern “*C. dacica*,” a stronger spatial autocorrelation was found, and the genetic neighborhood size was estimated to be 100–150 km. Although a major genetic cluster prevailed, some local genetic distinctiveness not interpreted as hybrids was evident within “*C. dacica*” (e.g., sites 3 Finse and 7 Memurudalen; Figure 2). As the origin and abundance of these local genetic groups are not known, it would be speculative to interpret them as separate seed‐sourcing regions based on our data. However, they do illustrate how using genomic methods to assess overall genetic structure and diversity can identify both larger seed‐sourcing regions and specific sites representing distinctive genetic variation.

Our choice of study system was not based on the suitability of *C. bigelowii* s.lat. for ecological restoration of alpine vegetation *per se*; although it has been recommended for the purpose, and is a well‐known study species in alpine ecology, it has a partially unresolved taxonomic complexity. We found two distinct evolutionary lineages that can be recognized morphologically, but our results also revealed widespread hybridization, potentially complicating a feasible use of *C. bigelowii* subsp. bigelowii or *C. dacica* for ecological restoration. Within each seed‐sourcing region, collecting seeds from fertile hybrids can, as far as possible, be avoided by identifying and recognizing intermediate genetic and/or morphological characteristics. Matching the habitats of the donor and restoration sites may further contribute to ensure a genetic match, as unknown small‐scale genetic differentiation between different habitats is found in many species (Vander Mijnsbrugge et al., 2010).

### 4.3 Conclusion and future perspectives

Parties to the Convention on Biological Diversity have acknowledged a taxonomic impediment to the sound management of biodiversity, and agreed to identify components of biological diversity important for its conservation and sustainable use (Article 7; CBD, 1992). The use of genomic tools in practical restoration ecology is still in its very beginning (Breed et al., 2019), and here we have shown how population genomics can alleviate the taxonomic impediment by offering a detailed genetic structure of a species complex, and inform practical questions and legal requirements related to improved local seed‐sourcing. Ideally, genetic and genomic data should be supplemented with common garden or reciprocal transplant experiments to further determine seeds most optimal for each ecological restoration site (De Kort et al., 2014), followed by further genetic assessment and monitoring with measures of genetic resilience of the restored populations (Thomas et al., 2014; Williams et al., 2014). Together with traditional taxonomic and ecological work, genomic information can enable the use of genetically appropriate seeds.

## CONFLICT OF INTEREST

None declared.

## AUTHOR CONTRIBUTIONS

**Kristine Bakke Westergaard:** Conceptualization (lead); Data curation (equal); Formal analysis (equal); Funding acquisition (lead); Investigation (lead); Methodology (equal); Project administration (lead); Resources (equal); Software (supporting); Validation (equal); Visualization (equal); Writing‐original draft (lead); Writing‐review & editing (lead). **Magni Olsen Kyrkjeeide:** Conceptualization (equal); Data curation (equal); Formal analysis (supporting); Investigation (equal); Methodology (equal); Project administration (equal); Resources (equal); Software (supporting); Validation (equal); Writing‐original draft (equal); Writing‐review & editing (supporting). **Marie Kristine Brandrud:** Data curation (equal); Formal analysis (equal); Investigation (equal); Methodology (equal); Software (lead); Validation (equal); Visualization (equal); Writing‐original draft (equal); Writing‐review & editing (supporting).

## Supporting information

Supplementary Material

## Data Availability

Both datasets A and B were uploaded to the Dryad Digital Repository (http://dx.doi.org/10.5061/dryad.4b8gthtdr).

## APPENDIX A

### De novo assembly of loci, variant calling, and filtering of SNPs

The following gives a detailed description of reference construction, read mapping, variant calling, and filtering of the SNP datasets.

Raw sequences were converted and demultiplexed using *bcl2fastq* (https://support.illumina.com/sequencing/sequencing_software/bcl2fastq‐conversion‐software.html), that is. converting and demultiplexing BCL files to FASTQ files, and running *fastQC* (http://www.bioinformatics.babraham.ac.uk/projects/fastqc/) and *fastQ Screen* (http://www.bioinformatics.babraham.ac.uk/projects/fastq_screen/) for each of the resulting six part‐libraries resulting from the *bcl2fastq* demultiplexing. *FastQC* screens the quality of the libraries and *fastQ Screen* runs the sequences against a set of sequence databases (Human, Rat, Mouse, PhiX and UniVec_Core) to check that the sequences match expectations, that is, no contamination. Overrepresented sequences were checked with BLAST (https://blast.ncbi.nlm.nih.gov/Blast.cgi). The six part libraries were further demultiplexed and quality checked separately with *process_radtags* 2.3e with settings to remove any read with an uncalled base, that is, [‐c], discard reads with low quality, that is [‐q] and rescue barcodes and RAD‐tags with one mismatch to a real sequence, that is. [‐r].

Loci were assembled with *denovo_map* 2.3e. The settings applied for the de novo assembly were chosen following the recommendations of parameter settings by Paris et al. (2017): for the parameter [‐m], that is, the number of reads needed to form a stack, it was suggested to test the range of [3–7]. The higher end of that range is recommended if the coverage is high; there is known or suspected contamination, and when conducting phylogenetic studies; otherwise, it is stated that the number of loci created is stabilizing after [‐m 3]. Since our study is not phylogenetic and the data were very clean, [‐m 3] and [‐m 5] were tested. Unless there is known high polymorphism or divergence in the dataset, the parameter [‐M], that is, the number of mismatches allowed between stacks within individuals, is further suggested best to be in the range of [2–3], and the parameter [‐n], that is, number of mismatches allowed between stacks between individuals, should be the same value as [‐M] ± 1. It is suggested to be at the higher end of that range when conducting phylogenetics, and at the lower end if samples were sampled from the same population. [‐M 2] and [‐M 3] were chosen to be tested and since this study neither is broadly phylogenetic nor sampled within the same population, n = M was chosen. *Splitstrees* (Huson & Bryant, 2005) were drawn for [‐m 3 ‐M 2 ‐n 2] and [‐m 5 ‐M 3 ‐n 3] to check for topological difference.

It is a common practice in population genetic analyses to filter out singletons, as they can potentially be contamination, sequencing errors or true rare loci, the latter of which would be considered noninformative in most population genetic analyses. Such loci are filtered out with minor allele frequency (MAF) or by restricting the amount of missing data allowed, although this is debatable since one might also be losing true and valuable information by doing so (Huang & Knowles, 2014; Linck & Battey, 2019). Linck and Battey (2019) found that model‐based analyses to infer population structure is strongly affected by the MAF choice, whereas nonmodel‐based analyses are less sensitive, and recommend to supplement model‐based analyses with nonmodel‐based analyses. Potentially wrongly merged loci, where two different loci have been merged to be alleles in one loci, would be expected to occur as heterozygotes throughout the dataset. To exclude such potential loci [‐‐max‐obs‐het] was set to [0.8].

From the 387 M raw paired reads retrieved in our study, 328 M were retained after demultiplexing and quality controls (see Table S1 for numbers of raw and retained reads per individual). On average, there were 60% duplicated reads and a GC content of 40%. *FastQ Screen* found no hits to human, rat, mouse, bacteria, or vector genomes, indicating clean data with little or no contamination. The *fastQC* reports looked as expected for RADseq data (there is inherent duplication and a barcode present). The reports picked up 74 overrepresented sequences, of which 64 had the best BLAST hit with the plastid genomes of different *Carex* species, three other had best BLAST hit to plastid genomes of other plants. These plastid loci could be retraced as 14 different loci in the catalog from the de novo assembly; however, none of these overrepresented sequences made it through to the final analyses because of the filters applied. There was no topological difference between [‐m 3 ‐M 2 ‐n 2] and [‐m 5 ‐M 3 ‐n 3], but higher number of SNPs and loci for [‐m 3 ‐M 2 ‐n 2]; thus, this setting was chosen (data not shown). The average coverage per individual was 27.4X.

### MAF sensitivity analyses

We ran a sensitivity analysis to illustrate the effect of the minor allele frequency filter (MAF) on the downstream analysis using sNMF (10 independent runs per *K* for *K* = 1–10) with MAF values 0.01, 0.05, 0.1, and 0.3. By looking for the “elbow” point on the resulting curves of the cross‐entropy criterion, *K* = 2, *K* = 3, and *K* = 4 were chosen to be displayed for the sensitivity analysis.

The first difference was seen in the cross‐entropy values (Figure S1), but although the values changed, the form of the curve did not change dramatically. Most likely, *K* (or range of most likely *K* values) was inferred consistently using different MAF values.

The second difference was seen in the affiliation of individuals to groups in the different runs (Figures S2–S4): different variants and different probabilities of the variants were found, where the groups suggested for the highest MAF value (0.3) were most divergent. For *K* = 2, there was no variation among runs, and all individuals were always affiliated to the same two groups (variant α). For *K* = 3 and *K* = 4, there were variations in the group affiliations. For *K* = 3 MAF 0.01, 0.05 and 0.1, two variants (β and γ) occurred, but their probabilities changed from variant β being most probable for MAF 0.01 to being least probable for MAF 0.05 and 0.1. A third variant (δ) was visible for MAF 0.3. For *K* = 4, altogether six variants occurred, one of them only for MAF 0.01, and three only for MAF 0.3.

By altering the MAF setting, in this case, increasing rare alleles get filtered out and the pattern of genetic structure recognized with MAF 0.01 became weaker until it was completely changed with MAF 0.3. The distinctiveness of the outgroup species *Carex nigra*, only represented by a few individuals, seemed to become tuned down when applying higher MAF values. Manipulation of data by applying higher MAF values can be done for various reasons, but should not be done naively, since it can change the overall structure and interpretation of the genetic data. For instance, an excess of common alleles could be interpreted as a sign of a population bottleneck, or absence of population subdivision, while high frequencies of rare alleles could be evidence of range expansion (Linck & Battey, 2019). Our choice of MAF value was intended to allow filtering out alleles only occurring in one individual, thus likely being a sequencing error and/or not biologically informative, and to keep rare alleles occurring in more than one individual (thus having an allele count >2, since one individual can be homozygote for one allele).
