# Supplementary material for: Using genomics to guide seed‐sourcing at the right taxonomical level for ecological restoration projects: The complex case of Carex bigelowii s.lat. in Norway
Source: Ecol Evol. 2021 Nov 17;11(23):17117–31. doi: 10.1002/ece3.8350 (PMC8668773; doi:10.1002/ece3.8350)
Supplement: Supplementary file 1 — Supplementary Material [file ECE3-11-17117-s001.docx]

**Supporting information**

**Using genomics to guide seed-sourcing at the right taxonomical level for ecological restoration projects: the complex case of *Carex bigelowii* s.lat. in Norway**

**Supporting figures and tables**

Figure S1. Cross-entropy criterion for *K*=1-10 performed with sNMF, MAF sensitivity analyses.

Figure S2. Bar plots from 10 sNMF runs for *K*=2, MAF sensitivity analyses.

Figure S3. Bar plots from 10 sNMF runs for *K*=3, MAF sensitivity analyses.

Figure S4. Bar plots from 10 sNMF runs for *K*=4, MAF sensitivity analyses.

Figure S5. Bayesian Information Criterion (BIC) plot.

Figure S6. DAPC scatter plot for *K*=3.

Figure S7. Mean likelihood probability plots summarized from STRUCTURE runs for *K*=1-4.

Figure S8. Bar plot showing the results of *K*=3 from STRUCTURE analyses.

Figure S9. Plot of cross-validation scores for *K*=1-10 performed with TESS3.

Figure S10. Bar plot showing the results of *K*=3 from TESS3 analyses.

Figure S11. Interpolated membership coefficients projected onto a map for *K*=3 from TESS3 analyses.

Figure S12. Histogram of test significance values resulting from the estimate of population differentiation for each loci compared to the genome-wide background performed using TESS3.

Figure S13. Manhattan plot for the -log10 p-values from the genome scan performed by TESS3.

Figure S14. Separate sNMF clustering analyses of site 33 Båtsfjord.

Figure S15. Two-dimensional PCoA plot (first and third axes), *Carex* *bigelowii* s.lat and *C. nigra*.

Figure S16. Heatmap visualising pairwise *F*_ST_ values, Norwegian sites of *Carex bigelowii*.

Figure S17. Cross-entropy criterion from sNMF analyses.

Table S1. Number of raw and retained reads after quality control.

Table S2. Pairwise comparisons of taxon-specific SNPs.


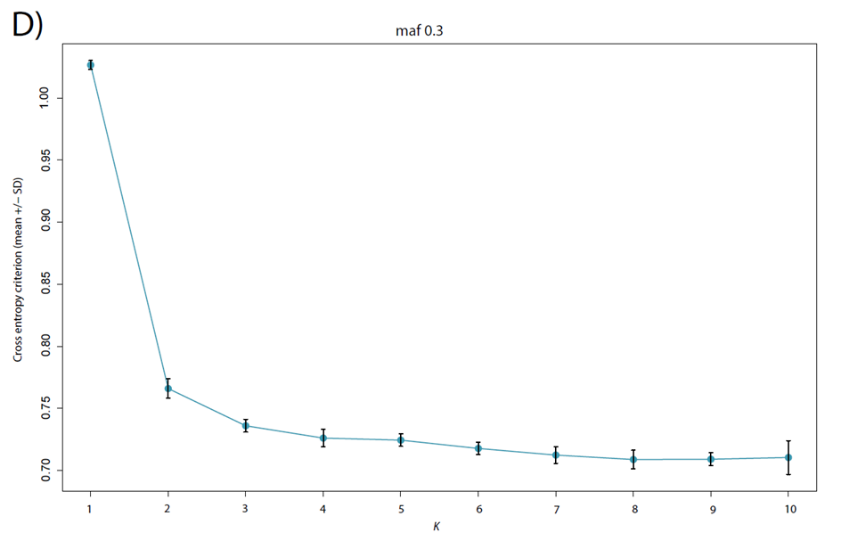

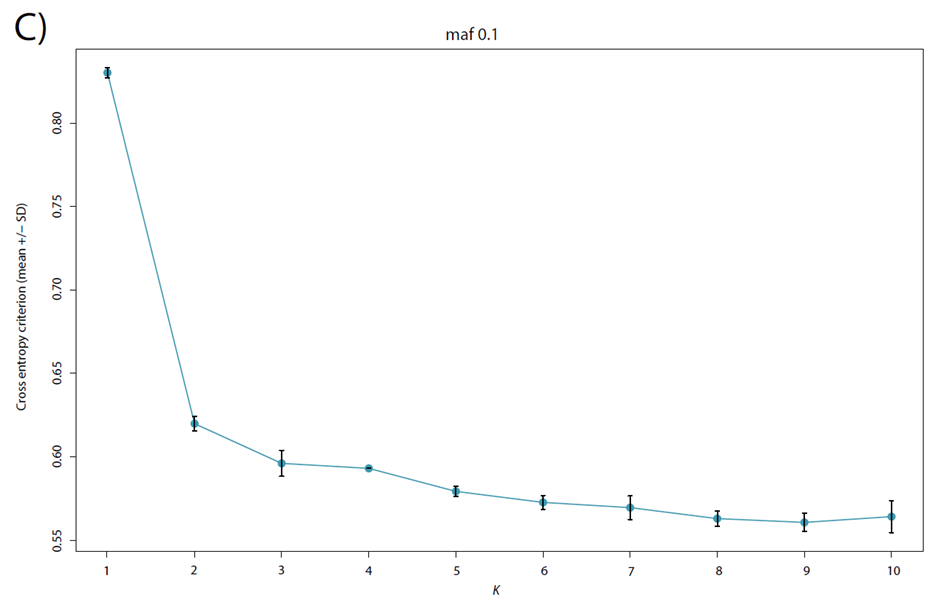

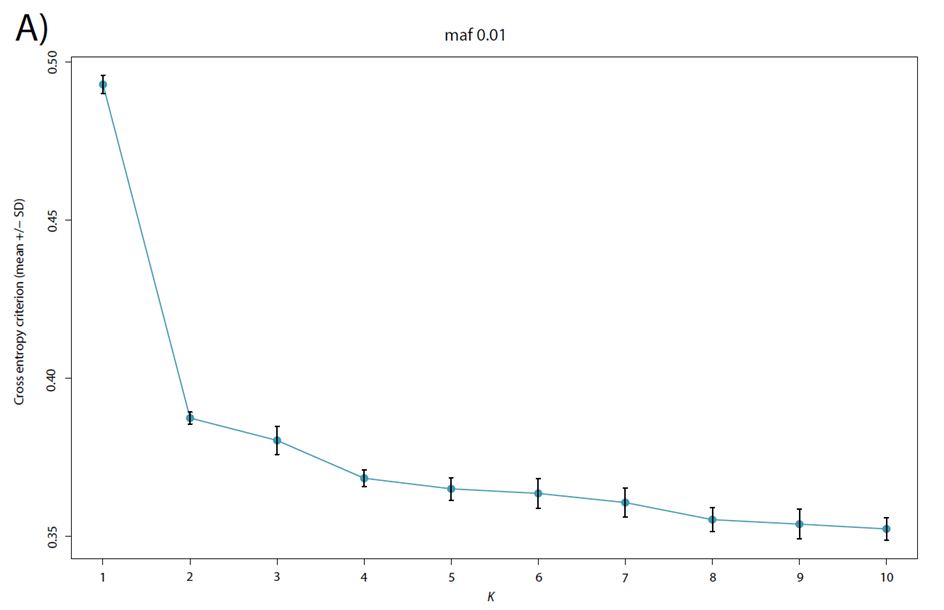

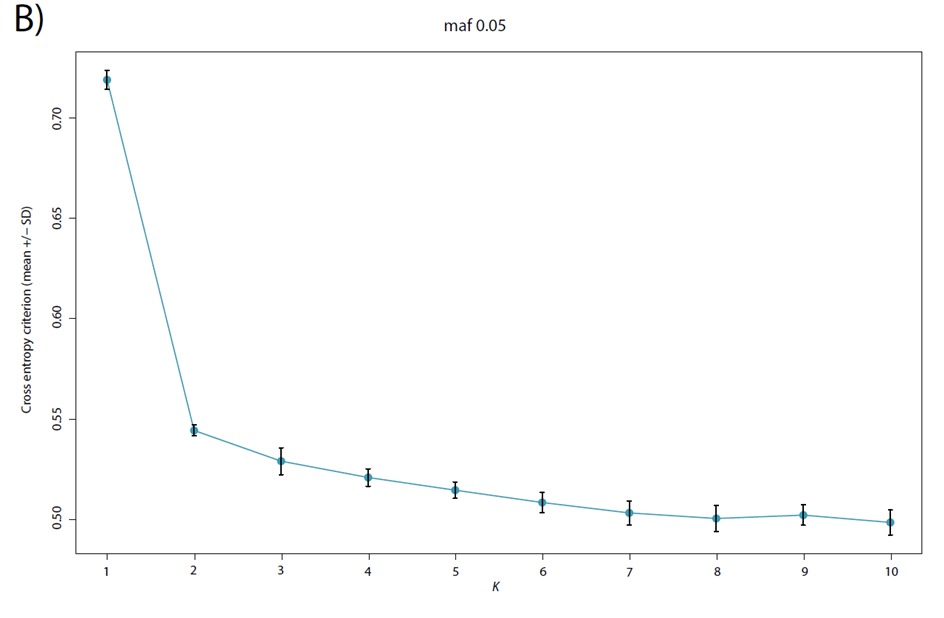


Figure S1. Cross-entropy criterion for *K*=1-10 performed with sNMF for 133 *Carex bigelowii* Torr. Ex Schwein s.lat. and outgroup *C*. *nigra* individuals (one replicate included; see Table 1 for information on individuals), 10 replicate runs: a) minor allele frequency filter of 0.01 (5,134 SNPs), b) minor allele frequency filter of 0.05 (2,876 SNPs), c) minor allele frequency filter of 0.1 (2,067 SNPs), d) minor allele frequency filter of 0.3 (663 SNPs).


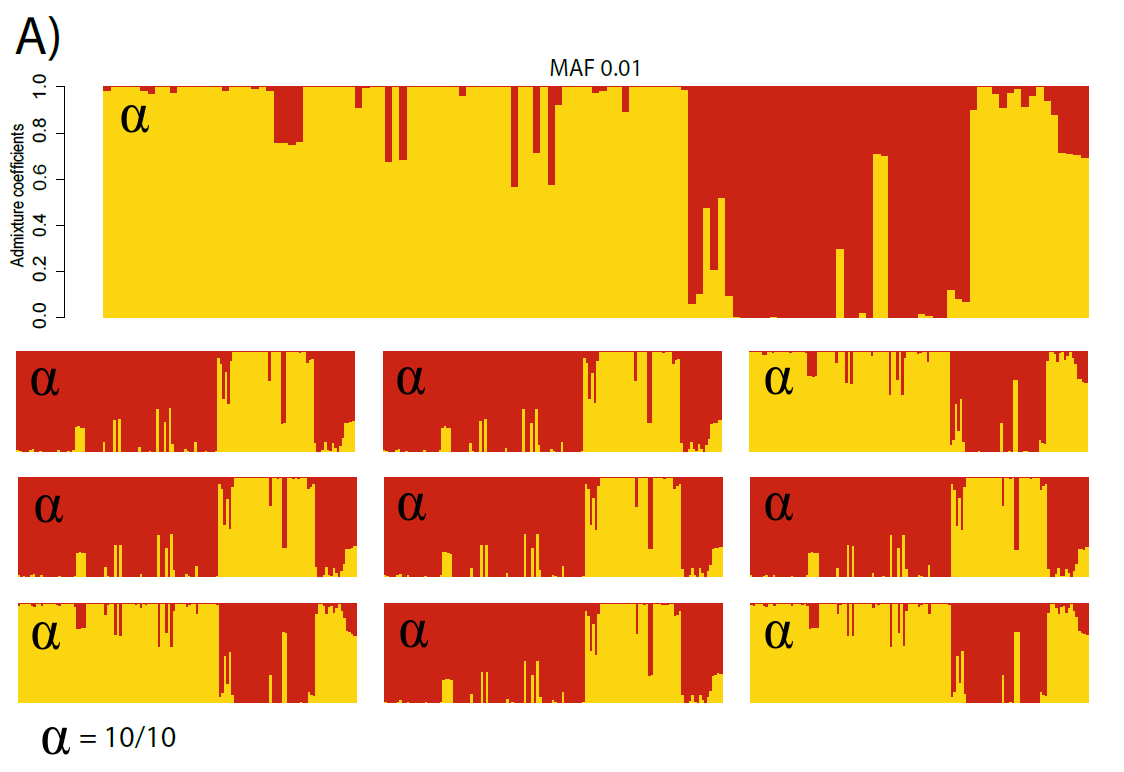

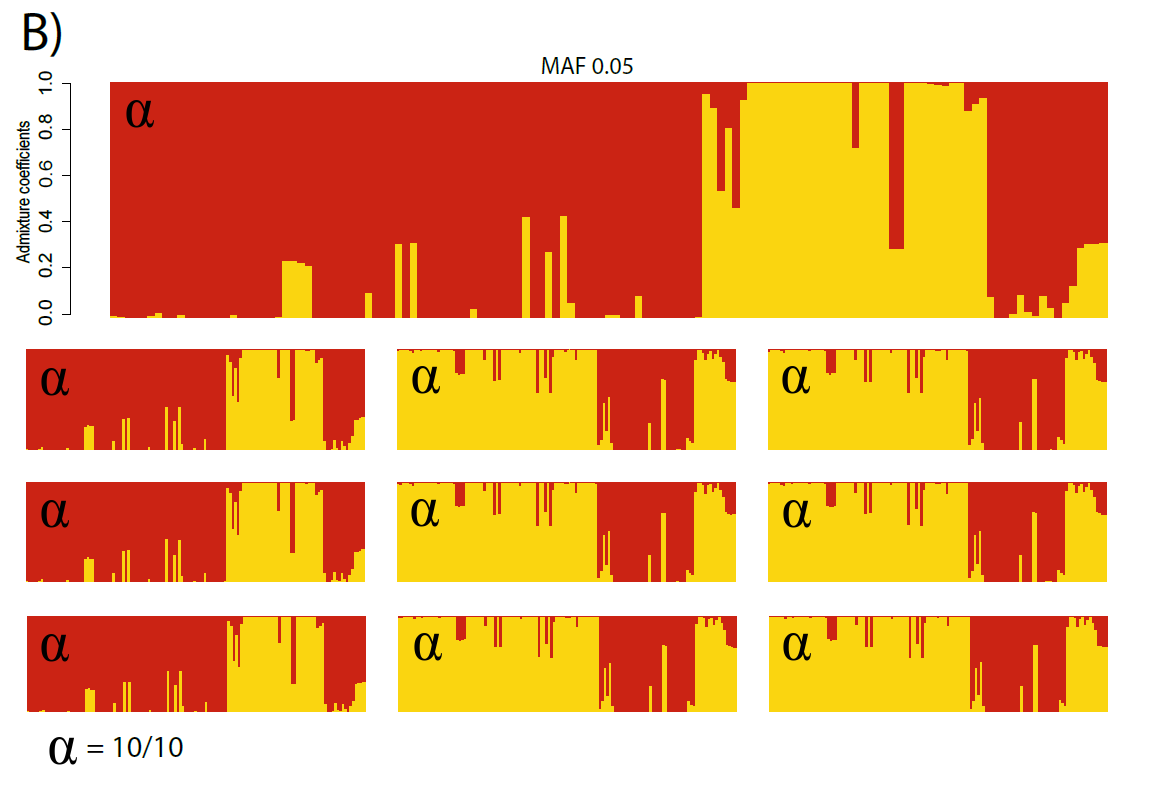


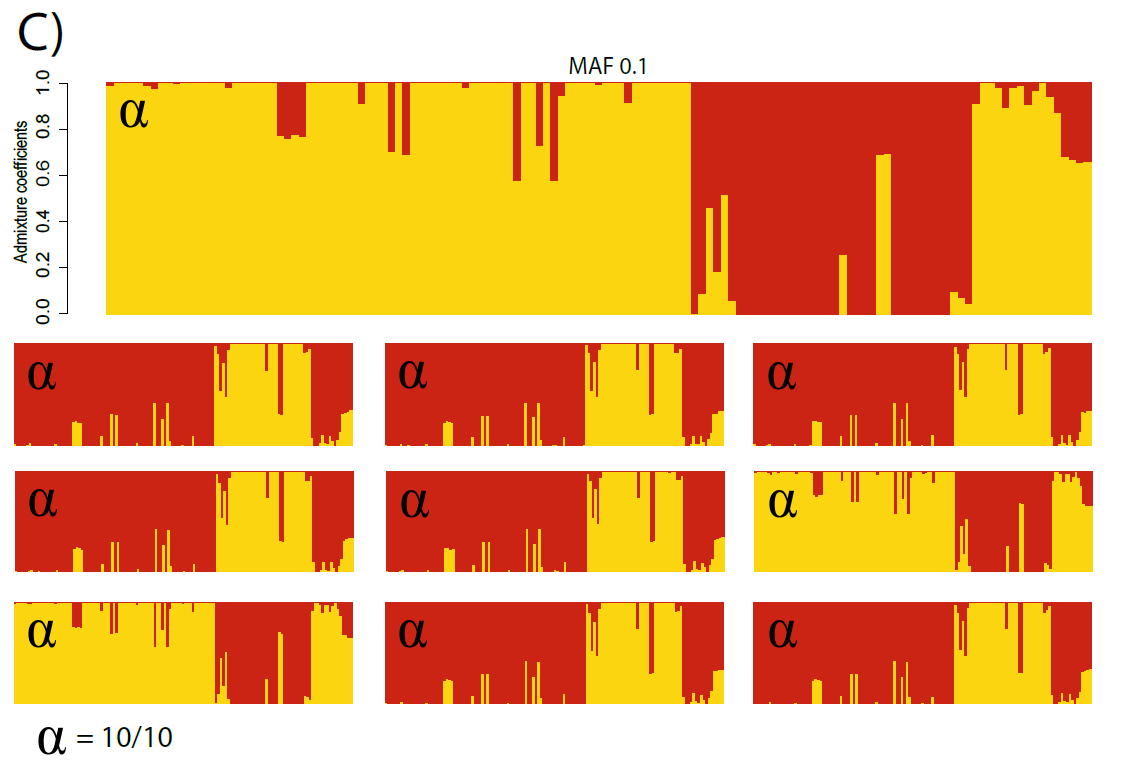


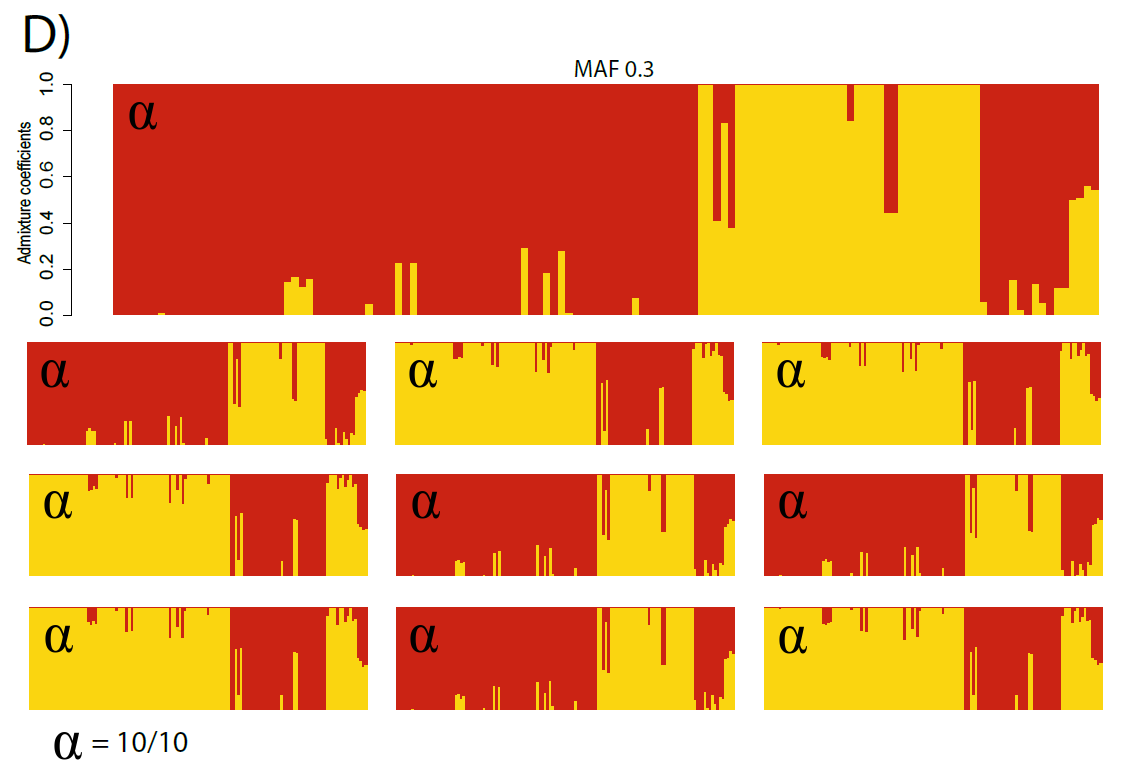


Figure S2. Bar plots from 10 sNMF runs for *K*=2, for 133 *Carex bigelowii* Torr. Ex Schwein s.lat. and outgroup *C*. *nigra* individuals (including one replicate; see Table 1 for information on individuals). The order of sites from left to right is the same as for the STRUCTURE bar plot (see Figure S6): a) minor allele frequency filter of 0.01 (5,134 SNPs), b) minor allele frequency filter of 0.05 (2,876 SNPs), c) minor allele frequency filter of 0.1 (2,067 SNPs), d) minor allele frequency filter of 0.3 (663 SNPs). Variants with a similar structure are marked with the same greek letter. The variants are summed up at the bottom of the figure.


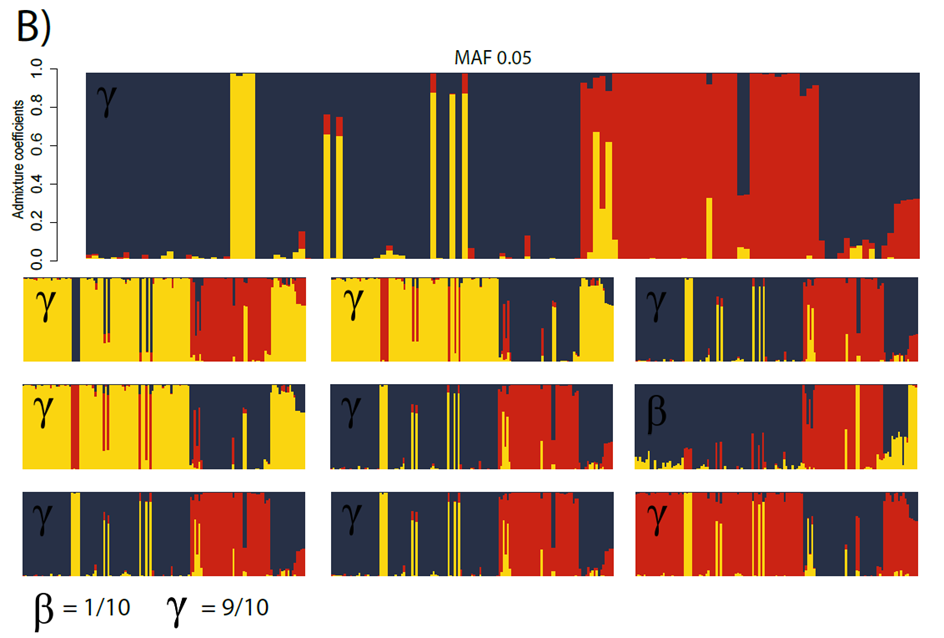

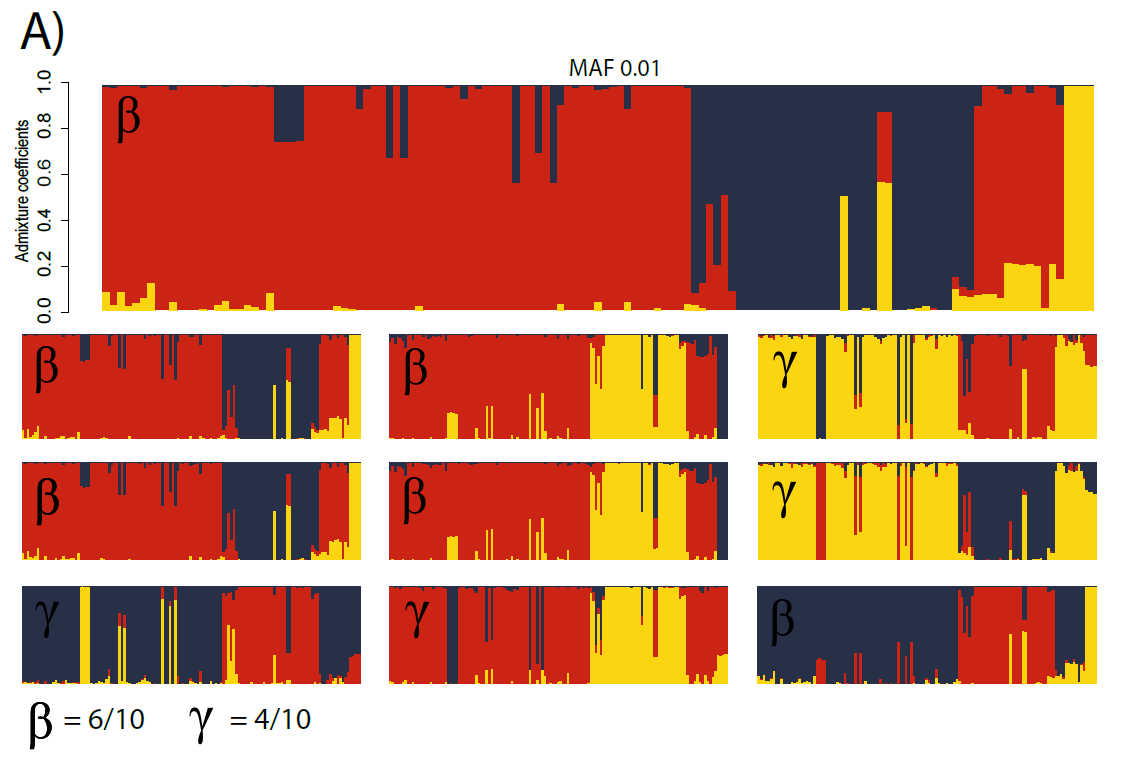


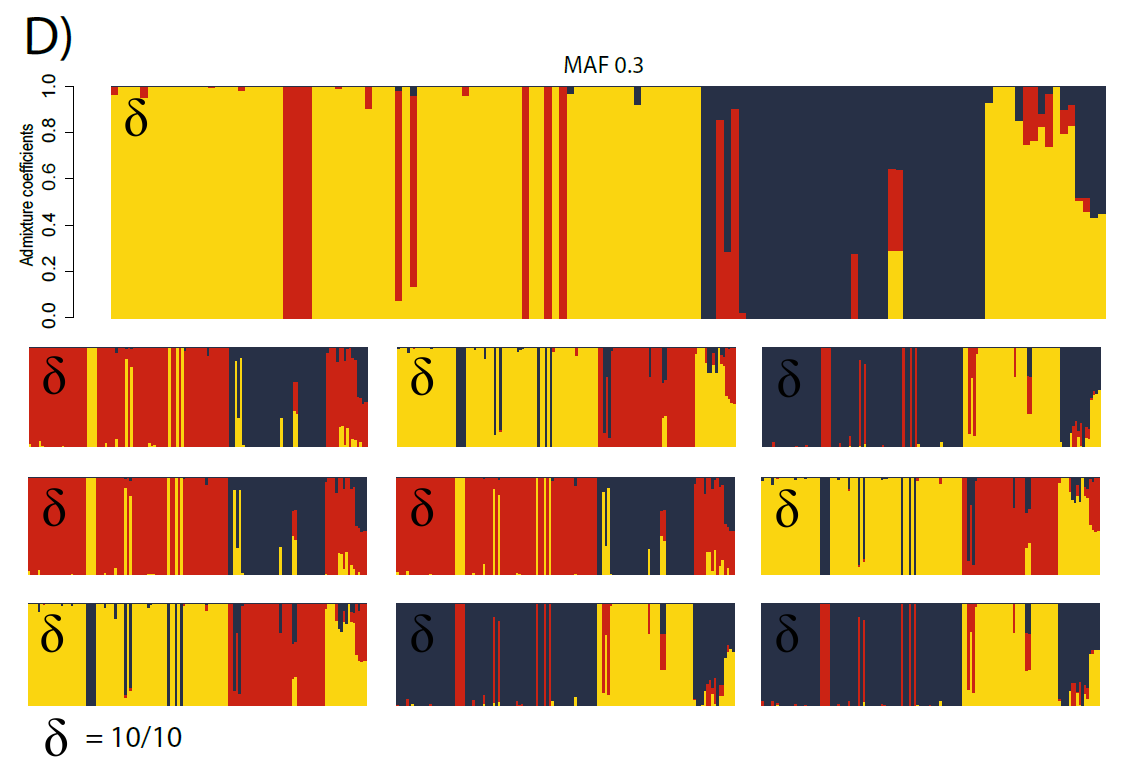

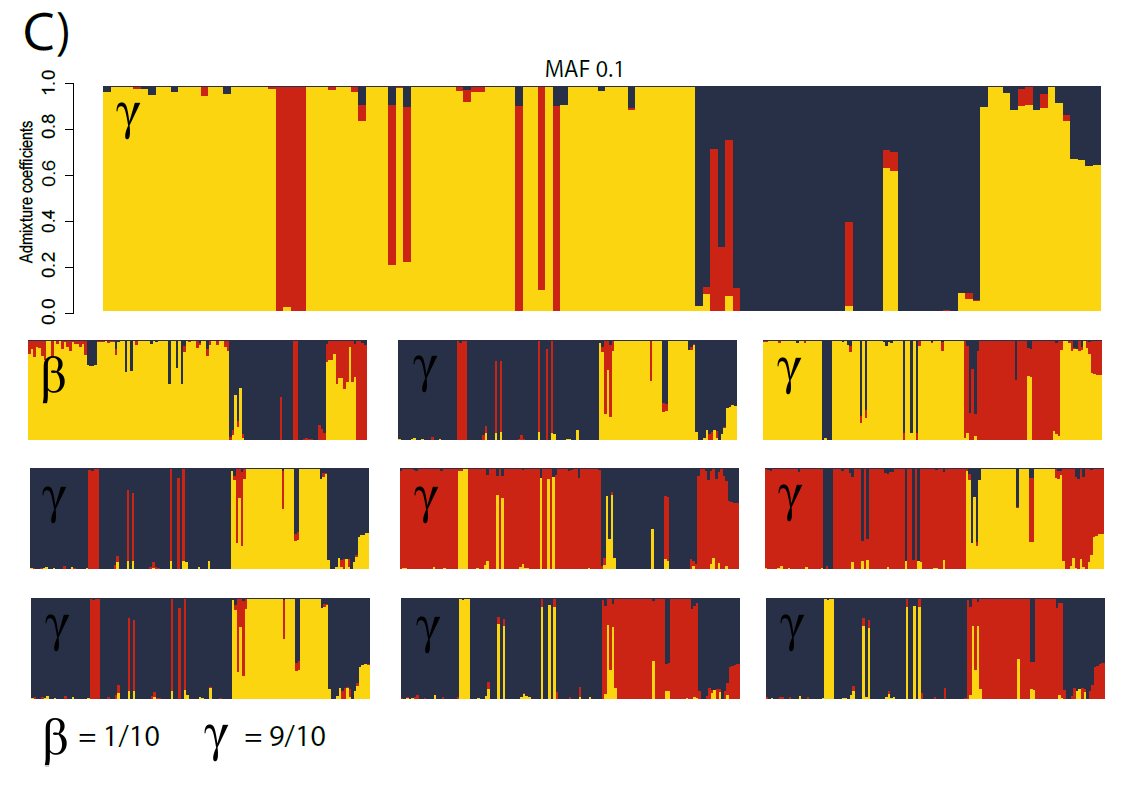
Figure S3. Bar plots from 10 sNMF runs for K=3, for 133 *Carex bigelowii* Torr. Ex Schwein s.lat. and outgroup *C*. *nigra* individuals (including one replicate; see Table 1 for information on individuals). The order of sites from left to right is the same as for the STRUCTURE bar plot (see Figure S6): a) minor allele frequency filter of 0.01 (5,134 SNPs), b) minor allele frequency filter of 0.05 (2,876 SNPs), c) minor allele frequency filter of 0.1 (2,067 SNPs), d) minor allele frequency filter of 0.3 (663 SNPs). Variants with a similar structure are marked with the same greek letter. The variants are summed up at the bottom of the figure.


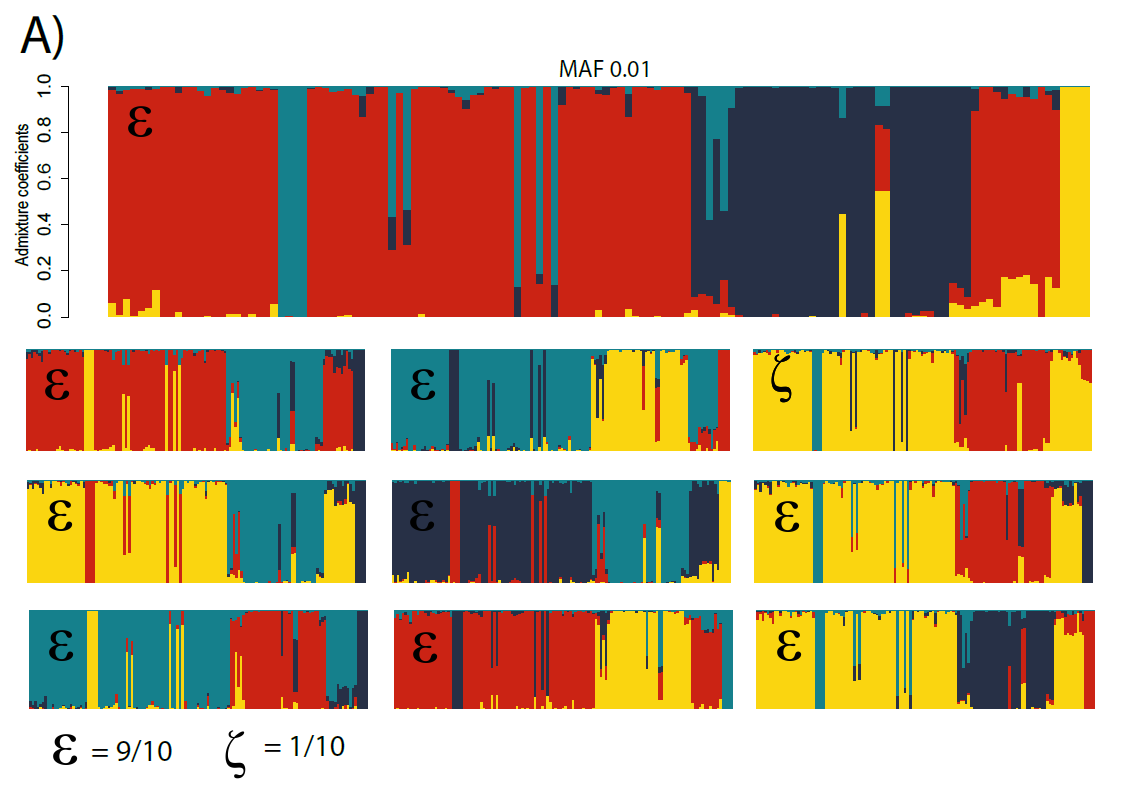

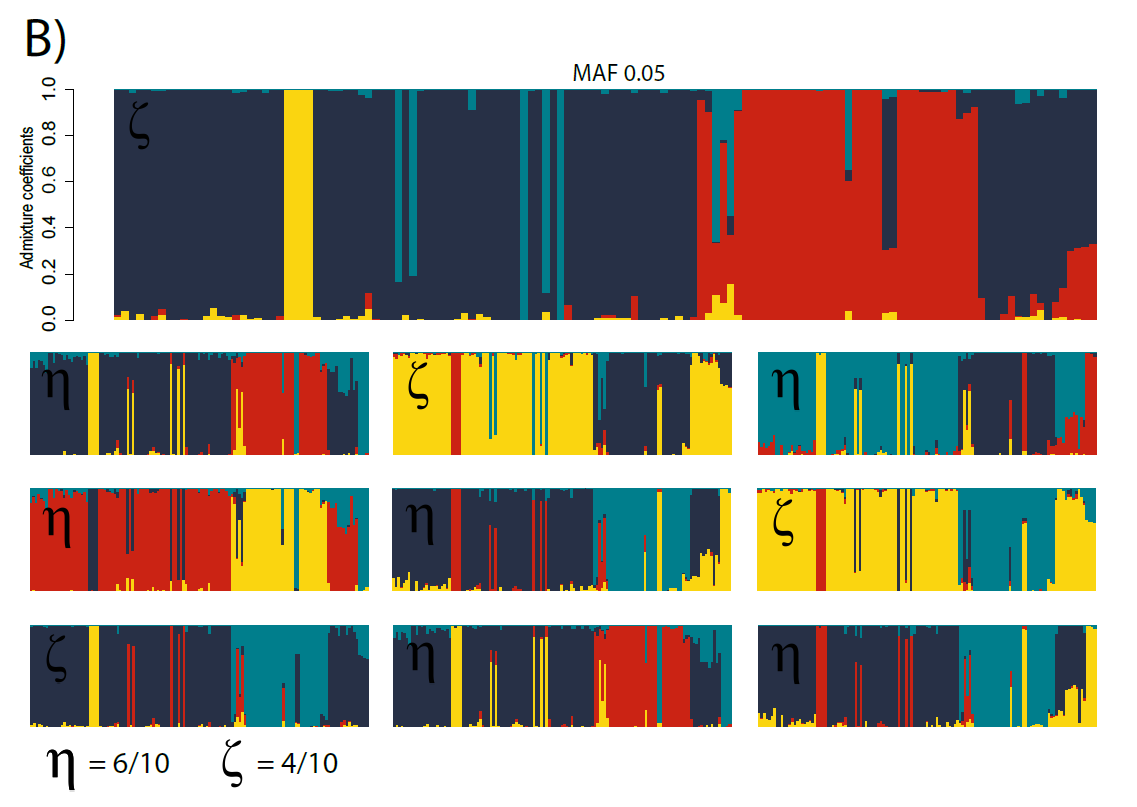


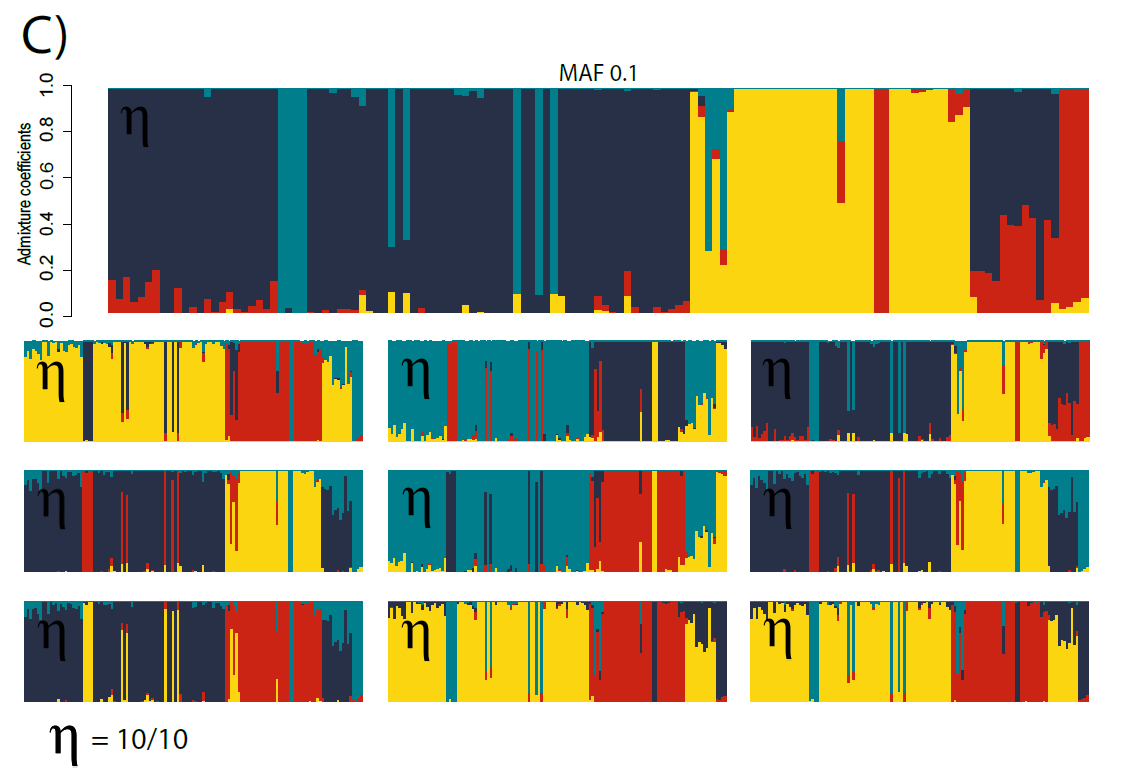

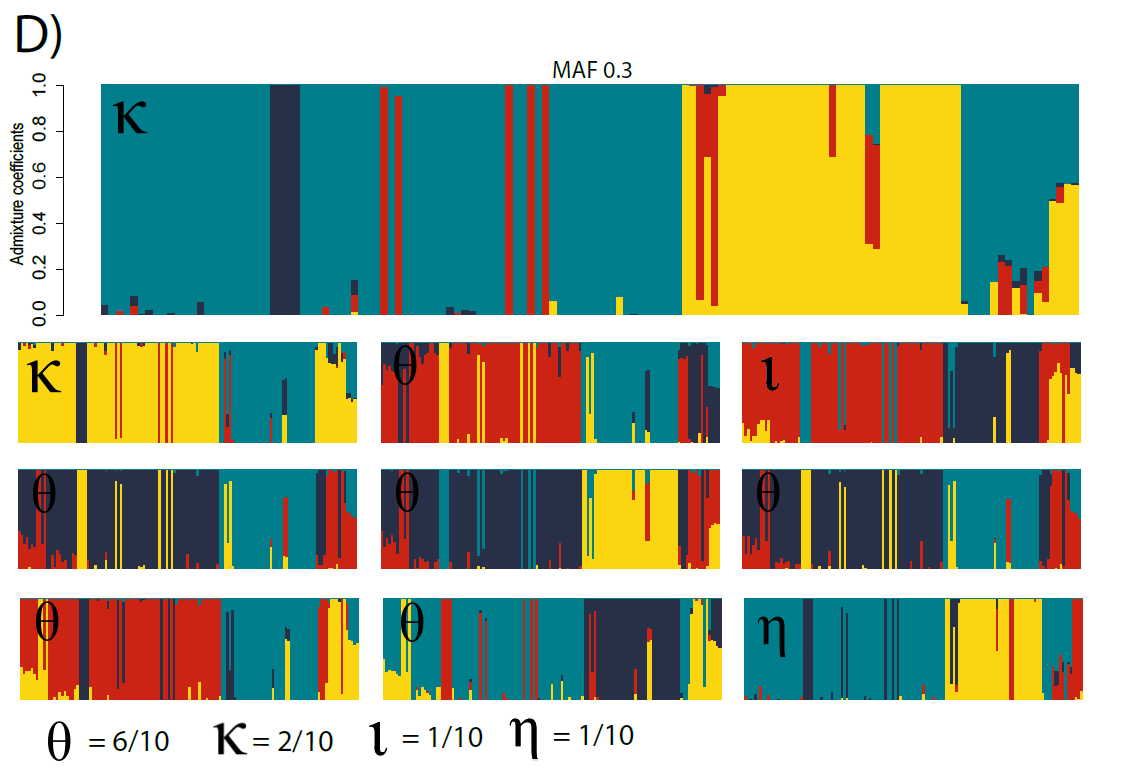
Figure S4. Bar plots from 10 sNMF runs for K=4, for 133 *Carex bigelowii* Torr. Ex Schwein s.lat. and outgroup *C*. *nigra* individuals (including one replicate; see Table 1 for information on individuals). The order of sites from left to right is the same as for the STRUCTURE bar plot (see Figure S6): a) minor allele frequency filter of 0.01 (5,134 SNPs), b) minor allele frequency filter of 0.05 (2,876 SNPs), c) minor allele frequency filter of 0.1 (2,067 SNPs), d) minor allele frequency filter of 0.3 (663 SNPs). Variants with a similar structure are marked with the same greek letter. The variants are summed up at the bottom of the figure.


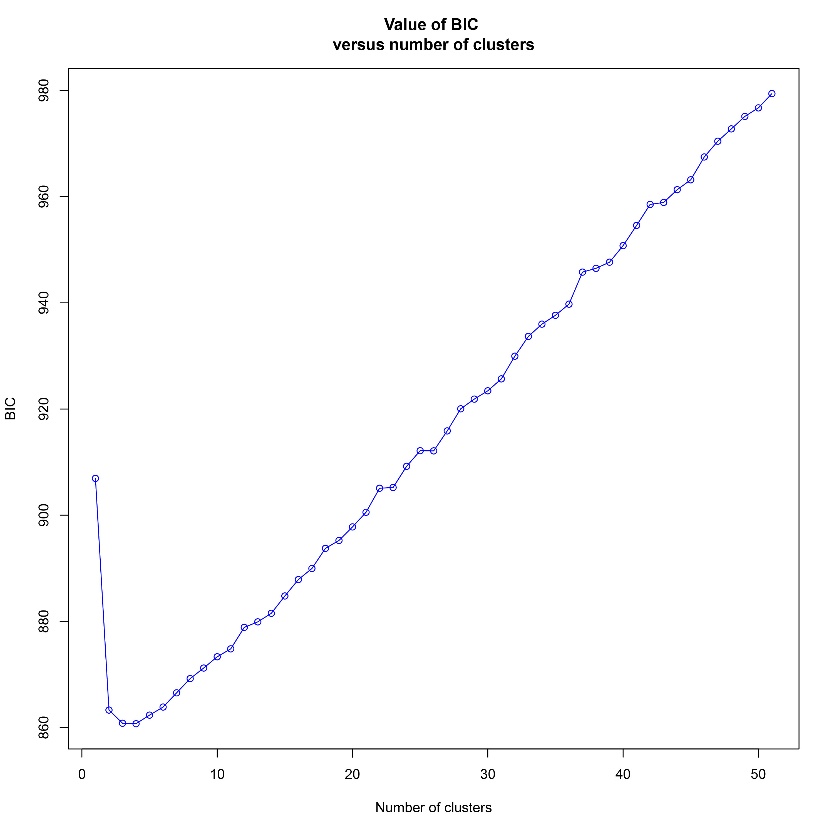


Figure S5. Bayesian Information Criterion (BIC) plot from *find.clusters()* analyses of all individuals of *C*. *bigelowii* s.lat. and *C*. *nigra* from 51 sites (Table 1). BIC value decreases until *K*=3, then begins to slightly increase at *K*=4-5, indicating three as the most likely number of clusters.


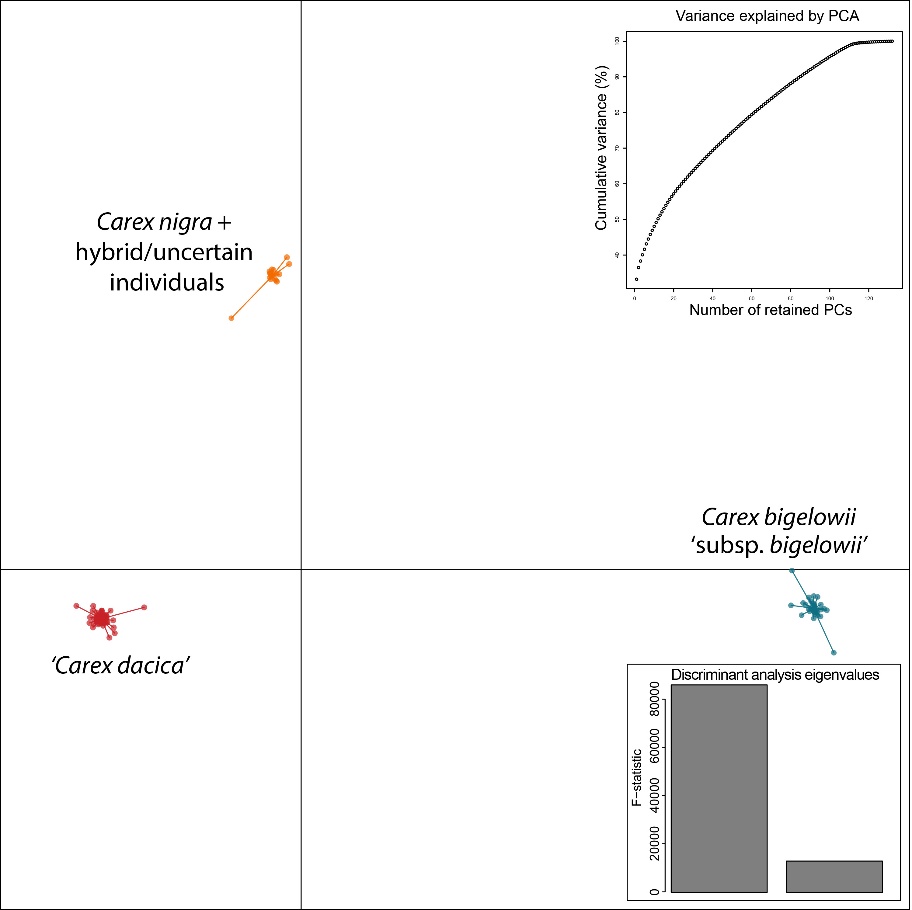


Figure S6. Discriminant analysis of principal component (DAPC) scatterplot of all individuals of *C*. *bigelowii* s.lat. and *C*. *nigra* from 51 sites (Table 1) using dataset A, with interpreted taxon names, and inset graphs of eigenvalues and variance explained by PCA.

Figure S7. Mean likelihood probability plots summarized from STRUCTURE runs for *K*=1-4 (performed with 1 million iterations and 100,000 burn-in, based on 5,134 SNPs) for all 132 *Carex bigelowii* s.lat. (including one replicate) and outgroup *C*. *nigra* individuals.


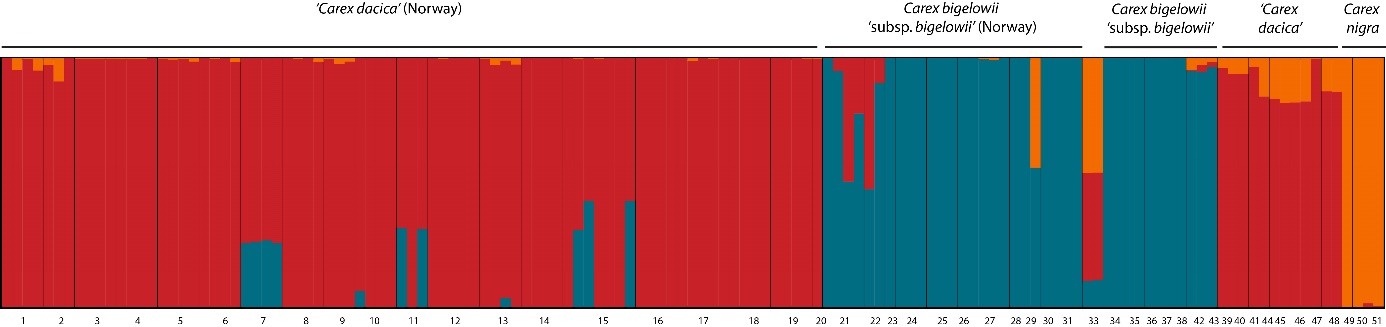


Figure S8. Bar plot showing the results of *K*=3 from STRUCTURE analyses performed with 1 million iterations and 100,000 burn-in, based on 5,134 SNPs of 132 individuals of *Carex* *bigelowii* s.lat (including one replicate) and outgroup *C. nigra*. Interpreted taxon names are given above, and site numbers are given at the bottom, see Table 1 for site information.


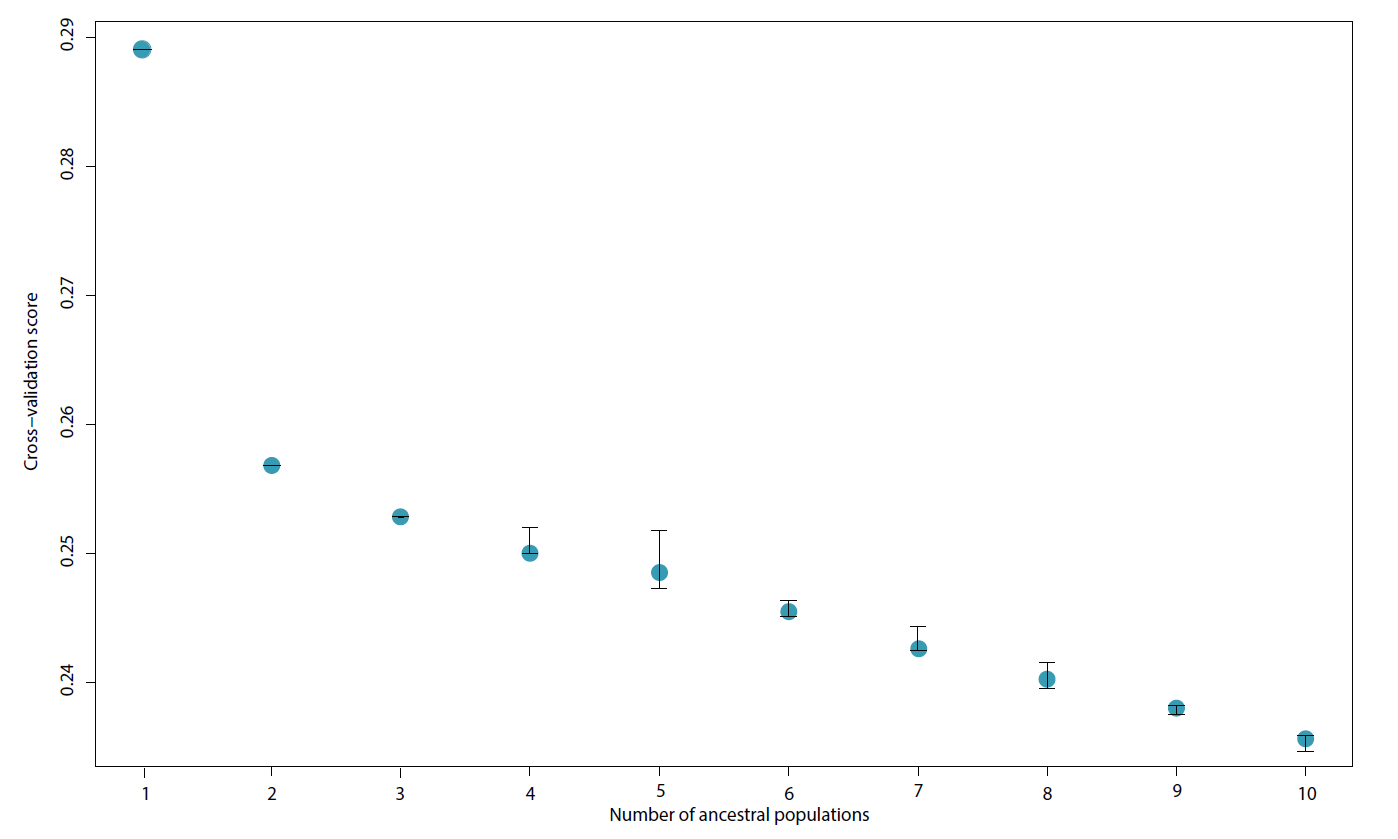


Figure S9. Plot of cross-validation scores for *K*=1-10 (10 replicate runs) performed with TESS3 for 132 *Carex* *bigelowii* s.lat. (including one replicate) and outgroup *C*. *nigra* individuals, based on 5,134 SNPs.


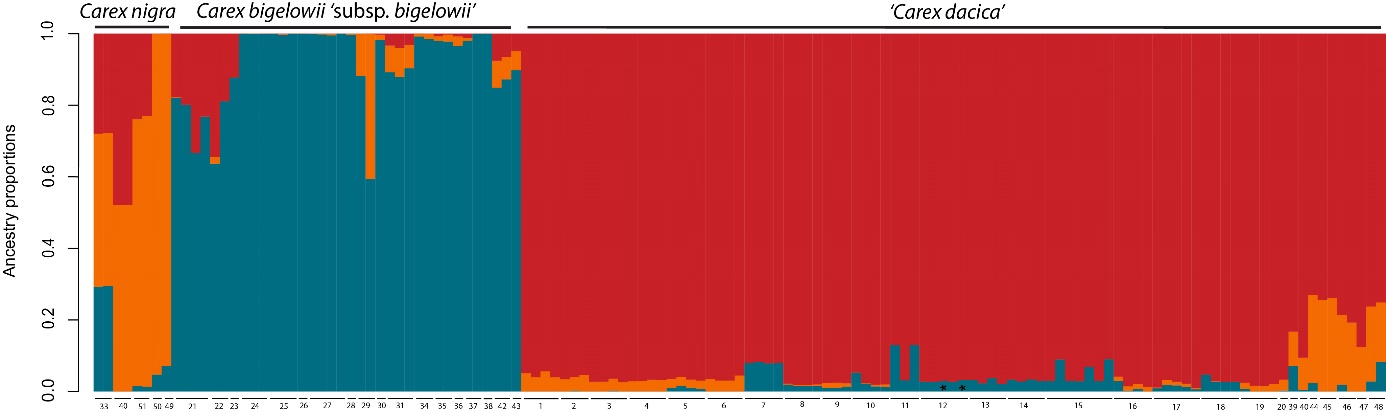


Figure S10. Bar plot showing the results of *K*=3 from TESS3 analyses (based on 5,134 SNPs) of 132 individuals of *Carex* *bigelowii* s.lat (including one replicate in site 12 marked with asterisks) and outgroup *C. nigra*. Individuals are ordered after a rough, interpreted taxonomic entity, site numbers are given at the bottom, see Table 1 for site information.


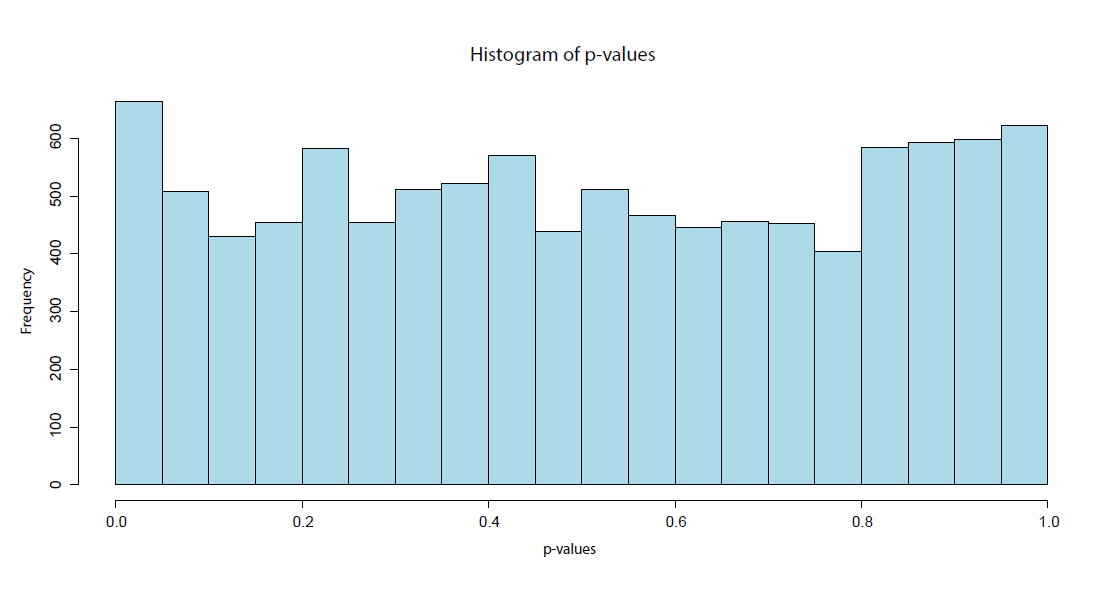

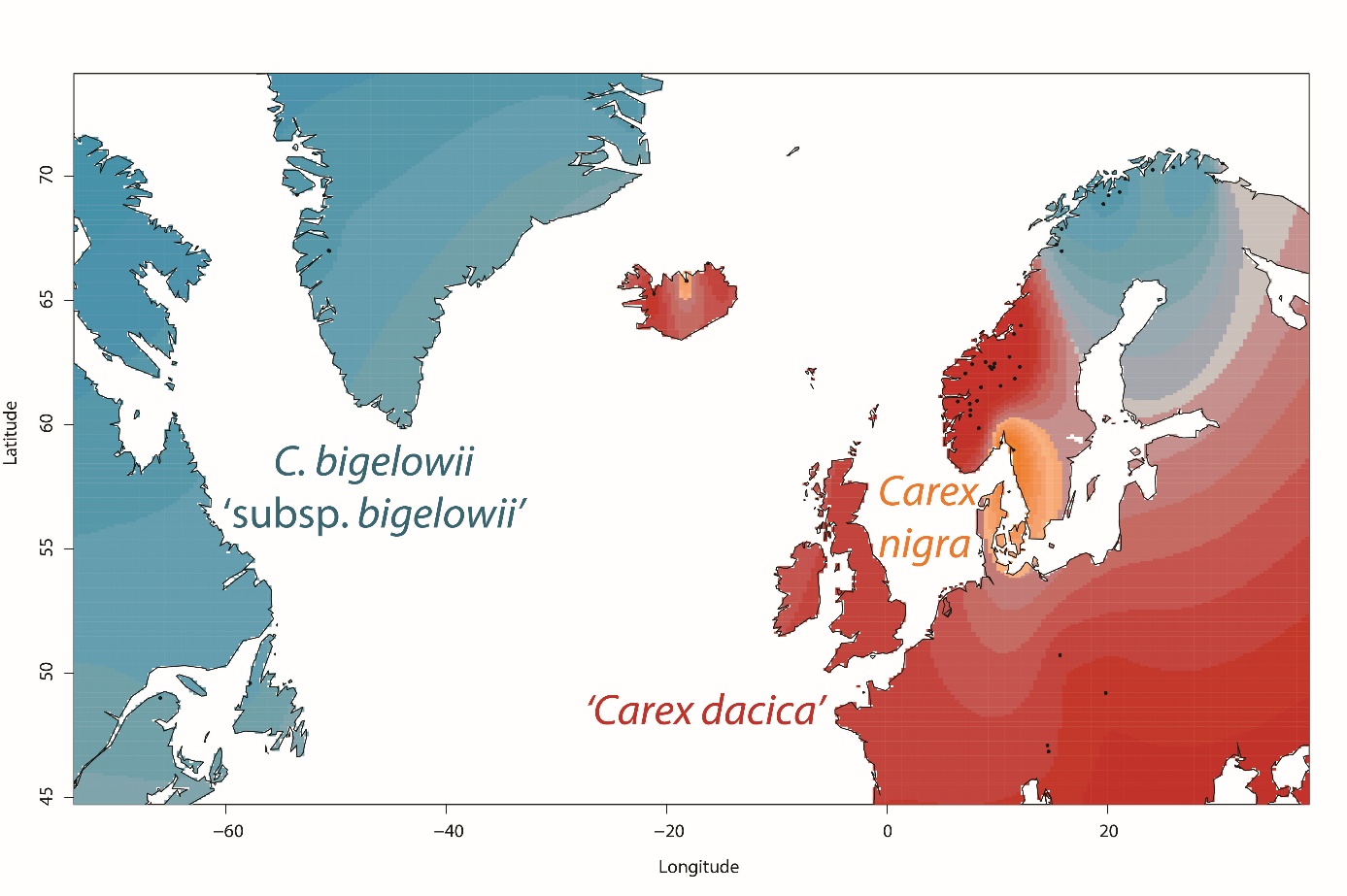
Figure S11. Interpolated membership coefficients projected onto a map for *K*=3 from TESS3 analyses (based on 5,134 SNPs) of 132 individuals of *Carex* *bigelowii* s.lat (including one replicate) and outgroup *C. nigra*, with interpreted taxonomic entities indicated for the blue group (*Carex bigelowii* ‘subsp. *bigelowii’*), the red group (‘*C*. *dacica’*) and the orange group (*C*. *nigra*). This map does not reflect the geographic distribution of the taxa.

Figure S12. Histogram of test significance values resulting from the estimate of population differentiation for each loci compared to the genome-wide background performed using TESS3 (based on 5,134 SNPs) for analyses of 132 individuals of *Carex* *bigelowii* s.lat (including one replicate) and outgroup *C. nigra*.


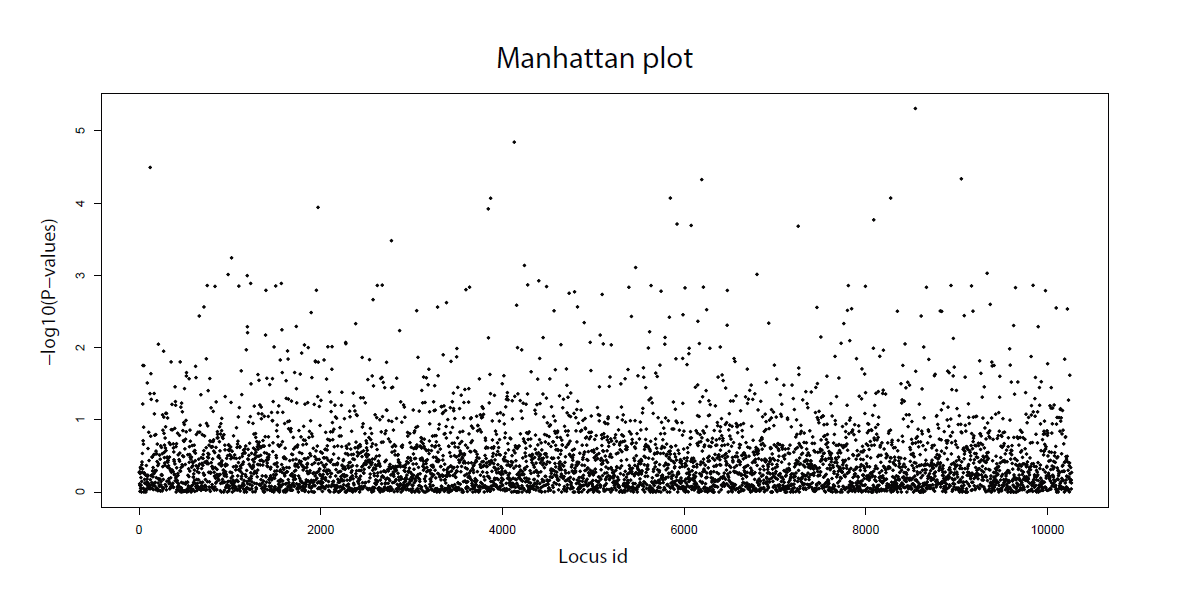


Figure S13. Manhattan plot for the -log10 p-values from the genome scan performed by TESS3 on 132 individuals of *Carex* *bigelowii* s.lat (including one replicate) and outgroup *C. nigra*. No candidate loci were found with a Benjamini-Hochberg corrected FDR control for the p value of 0.0001.


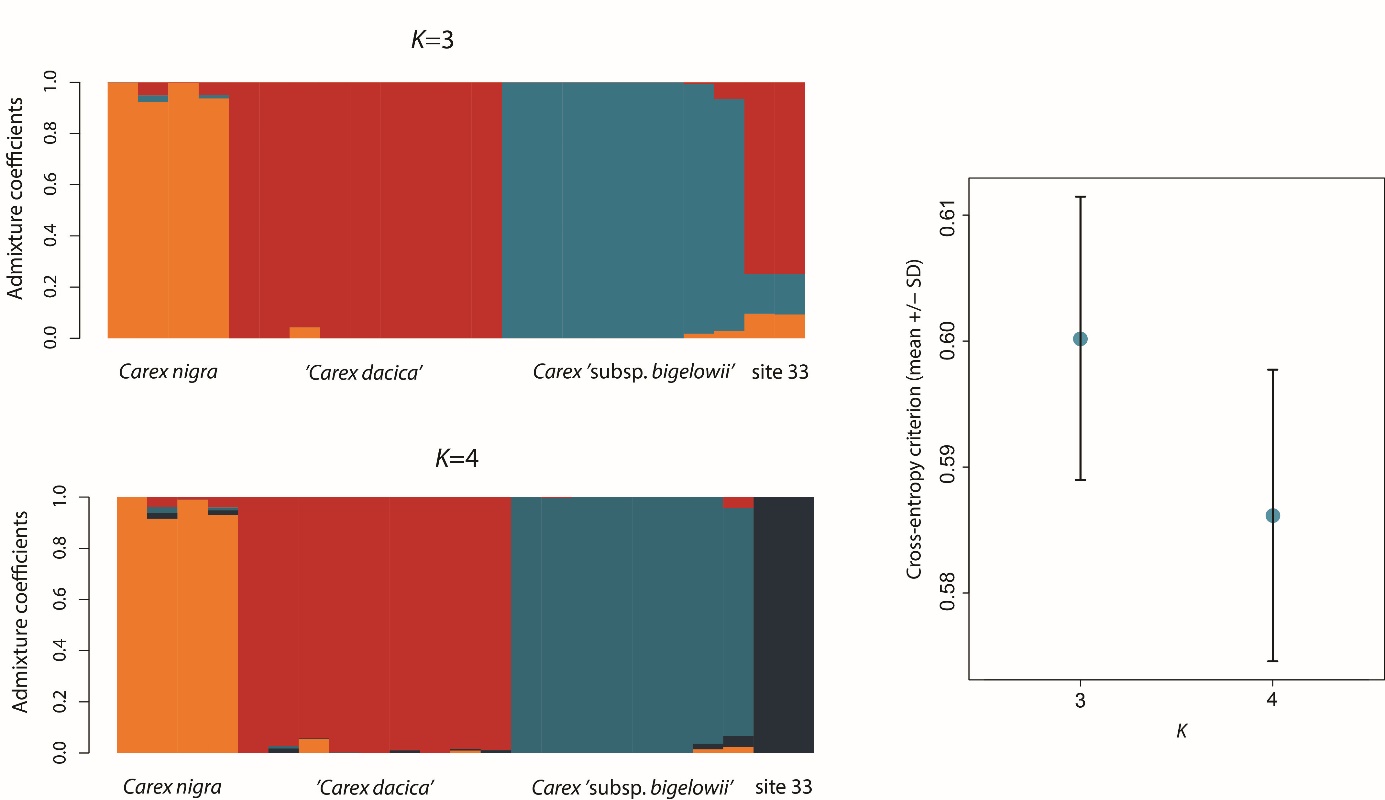


Figure S14. Separate clustering analyses of site 33 Båtsfjord performed with a sub-sample of individuals from the clusters *Carex nigra*, ‘*C*. *dacica*’ and *C*. *bigelowii* ‘subsp. *bigelowii*’ with sNMF for *K*=3 (top left) and *K*=4 (bottom left), and the summarized cross-entropy criterion for the respective runs (right). The analyses were performed with 5,134 SNPs and 10 replicate runs.


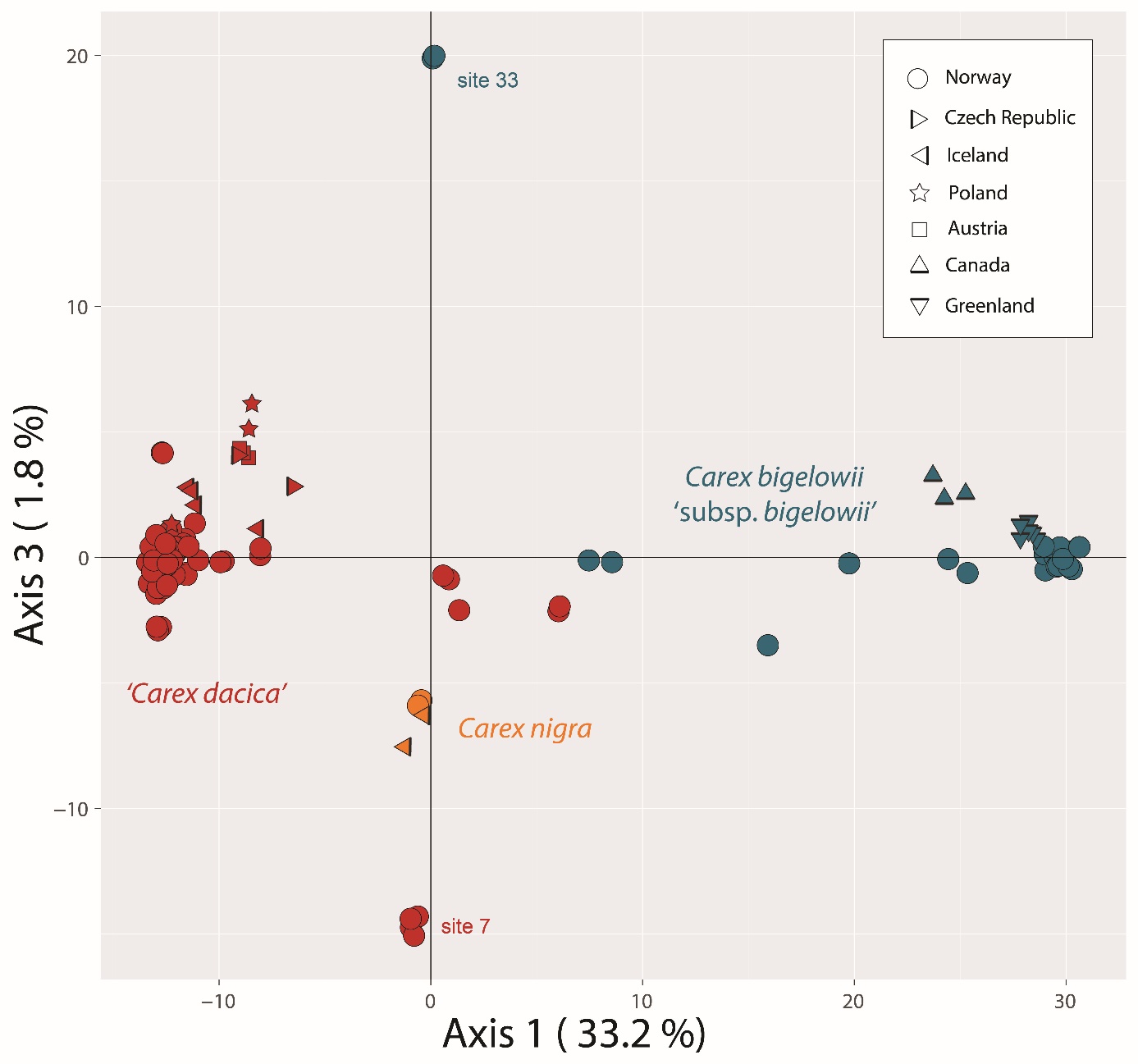


Figure S15. Two-dimensional PCoA plot (first and third axes) based on 15,095 SNPs in all 132 individuals of *Carex* *bigelowii* s.lat (including one replicate) and outgroup *C. nigra*, where the geographical origin of each individual is indicated with different shaped symbols. Individuals from two sites 7 and 33 are highlighted (see Table 1 for site information).


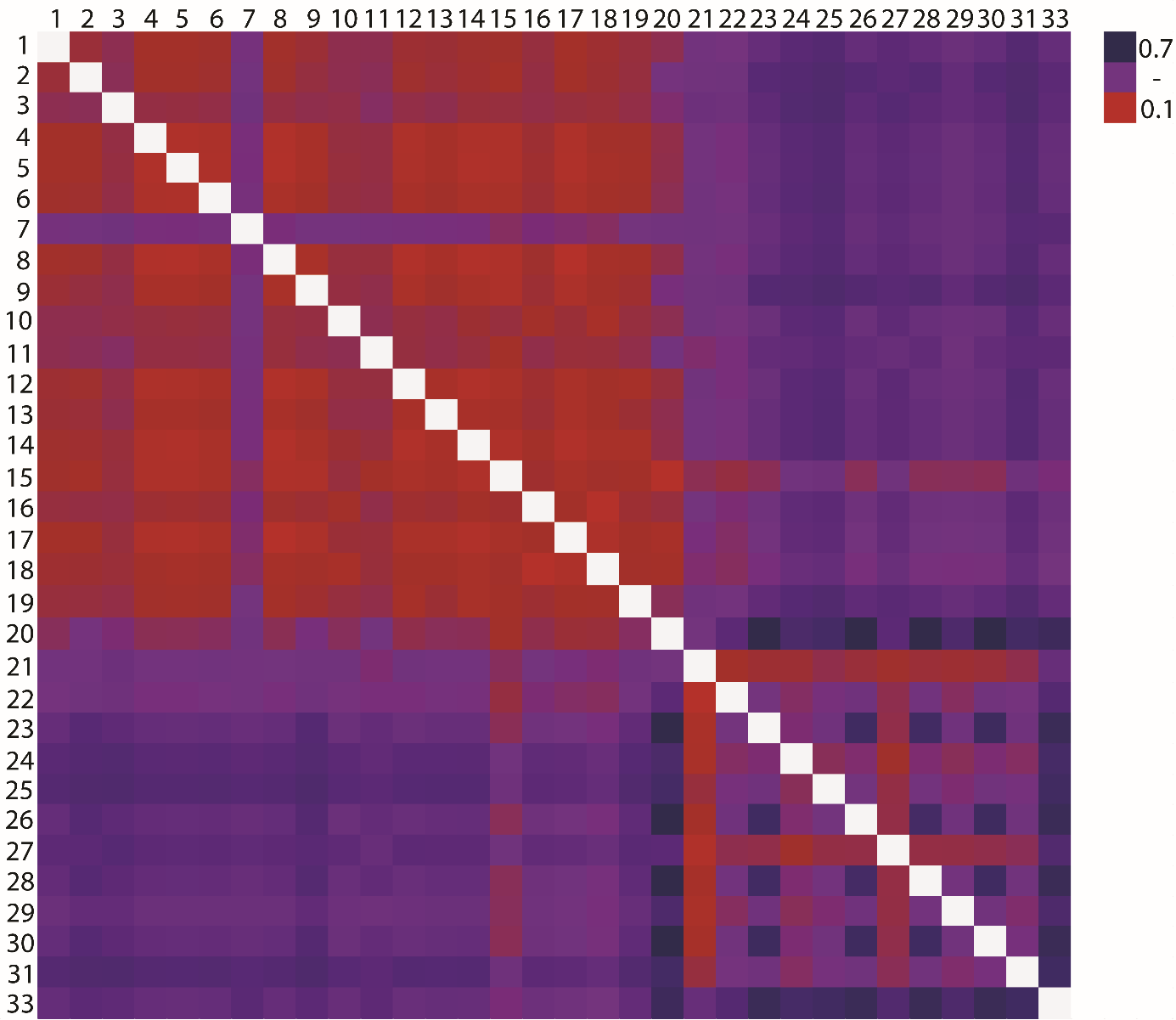


Figure S16. Heatmap visualising pairwise *F*_ST_ values (based on 5,134 SNPs) between 32 Norwegian sites of *Carex bigelowii* Torr. Ex Schwein s.lat., see Table S1 for site and individual information.


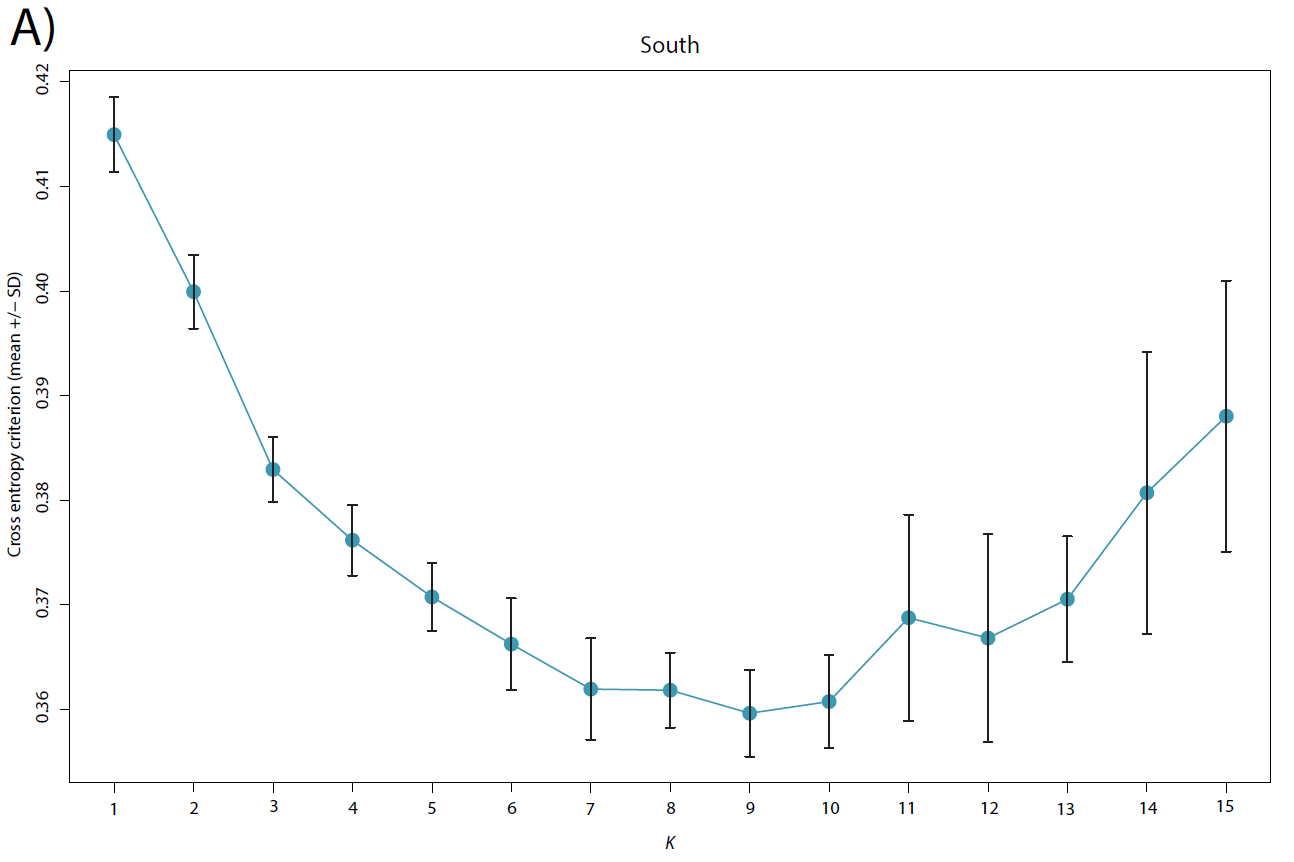

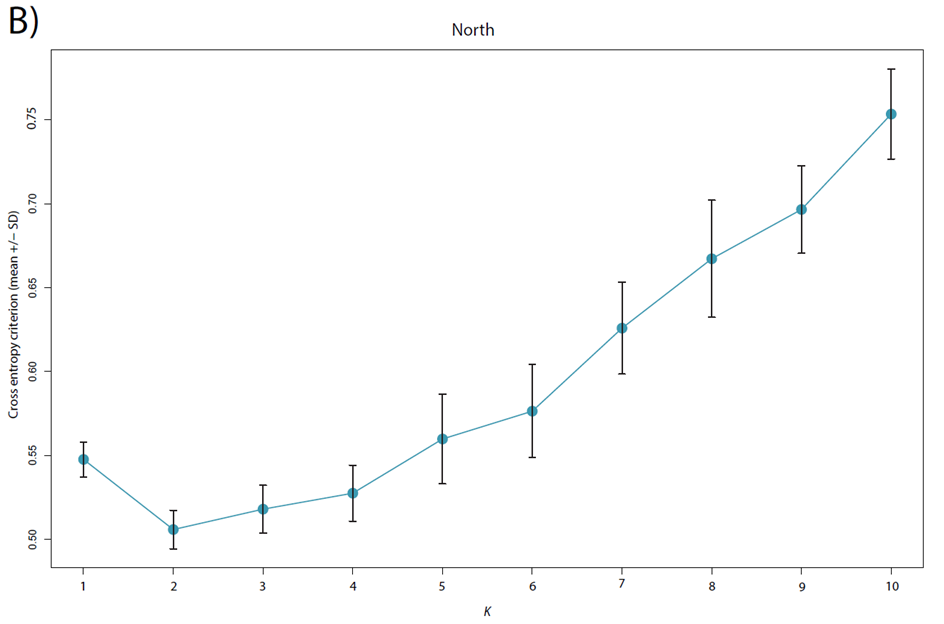
Figure S17. Cross-entropy criterion from sNMF analyses (5 134 SNPs, 10 replicate runs) for a) *K*=1-15 for 78 individuals and one replicate of ‘*Carex dacica*’ from southern Norway, b) *K*=1-10 for 27 individuals of *Carex bigelowii* ‘subsp. *bigelowii*’ from northern Norway.

Table S1. Number of raw and retained reads after quality control for for 132 *Carex bigelowii* Torr. Ex Schwein s.lat. and outgroup *C*. *nigra* individuals, including one replicate marked with an asterisk (see Table 1 for site information).

| Individual number | Site | Raw reads | Retained reads |
| --- | --- | --- | --- |
| 11_6 | 1 | 1825908 | 1790551 |
| 11_9 | 1 | 3501508 | 3428304 |
| 11_5 | 1 | 3904300 | 3781755 |
| 11_8 | 1 | 3144420 | 3089001 |
| 25_7 | 2 | 6090384 | 5846724 |
| 25_3 | 2 | 3302974 | 3255605 |
| 25_9 | 2 | 6858314 | 6750935 |
| 2_2 | 3 | 5281298 | 5201777 |
| 2_5 | 3 | 6411936 | 6304959 |
| 2_8 | 3 | 4605522 | 4531642 |
| 2_9 | 3 | 10738376 | 10563908 |
| 14_4 | 4 | 2739408 | 2642538 |
| 14_7 | 4 | 3096554 | 3007195 |
| 14_10 | 4 | 4191480 | 4121489 |
| 14_1 | 4 | 3232684 | 3182616 |
| 23_3 | 5 | 3251748 | 3202845 |
| 23_10 | 5 | 4725292 | 4637897 |
| 23_5 | 5 | 3753820 | 3680922 |
| 23_7 | 5 | 3181594 | 3134921 |
| 22_8 | 6 | 5146474 | 5052719 |
| 22_10 | 6 | 3138842 | 3083537 |
| 22_1 | 6 | 4910514 | 4814229 |
| 22_3 | 6 | 4225298 | 4163317 |
| 4_9 | 7 | 2219570 | 2176626 |
| 4_10 | 7 | 2314238 | 2262047 |
| 4_8 | 7 | 3910014 | 3848090 |
| 4_2 | 7 | 5725302 | 5546077 |
| 17_7 | 8 | 2322312 | 2279826 |
| 17_4 | 8 | 4558414 | 4484568 |
| 17_2 | 8 | 6169484 | 6082784 |
| 17_1 | 8 | 5630400 | 5533694 |
| 9_2 | 9 | 2583966 | 2535215 |
| 9_7 | 9 | 4515882 | 4425297 |
| 9_10 | 9 | 7083188 | 6956346 |
| 6_10 | 10 | 3888558 | 3827194 |
| 6_5 | 10 | 4614318 | 4538502 |
| 6_1 | 10 | 7616082 | 7454336 |
| 6_7 | 10 | 7714546 | 7590244 |
| 5_3 | 11 | 6730246 | 6636189 |
| 5_10 | 11 | 4145846 | 4010312 |
| 5_5 | 11 | 7311910 | 7171552 |
| 26_2 | 12 | 5572922 | 5497581 |
| 26_5 | 12 | 6396030 | 6254958 |
| 26_9* | 12 | 7888666 | 7743253 |
| 26_3 | 12 | 8155828 | 7974719 |
| 26_9* | 12 | 3210082 | 3159173 |
| 13_6 | 13 | 5230162 | 5126874 |
| 13_10 | 13 | 2544794 | 2507625 |
| 13_1 | 13 | 1983654 | 1948867 |
| 13_7 | 13 | 6986910 | 6817358 |
| 18_7 | 14 | 4364352 | 4299467 |
| 18_10 | 14 | 8172240 | 8043837 |
| 18_4 | 14 | 2936180 | 2867660 |
| 18_2 | 14 | 6961808 | 6837263 |
| 15_10 | 15 | 2887802 | 2835935 |
| 15_9 | 15 | 4431628 | 4356825 |
| 15_2 | 15 | 7198442 | 7083030 |
| 15_1 | 15 | 6785854 | 6673070 |
| 15_5 | 15 | 7939392 | 7781015 |
| 15_3 | 15 | 4456822 | 4378489 |
| 15_8 | 15 | 7980696 | 7863228 |
| 8_3 | 16 | 4985818 | 4902477 |
| 8_1 | 16 | 7061306 | 6814331 |
| 8_6 | 16 | 9529314 | 9352179 |
| 8_4 | 16 | 4155342 | 4058674 |
| 21_4 | 17 | 2324560 | 2285200 |
| 21_2 | 17 | 2370862 | 2323453 |
| 21_8 | 17 | 1379360 | 1350197 |
| 21_1 | 17 | 2928808 | 2881627 |
| 21_7 | 17 | 5352662 | 5199480 |
| 3_2 | 18 | 3702578 | 3639952 |
| 3_3 | 18 | 3572458 | 3513272 |
| 3_10 | 18 | 5743650 | 5640313 |
| 3_8 | 18 | 9808546 | 9606489 |
| 16_8 | 19 | 2094686 | 2060909 |
| 16_3 | 19 | 5718118 | 5608288 |
| 16_1 | 19 | 6300582 | 6207259 |
| 16_6 | 19 | 3888828 | 3825928 |
| 20_2 | 20 | 2347590 | 2307877 |
| 19_1 | 21 | 3409200 | 3349554 |
| 19_5 | 21 | 2215892 | 2019189 |
| 19_3 | 21 | 2929010 | 2872934 |
| 19_10 | 21 | 3571858 | 3510795 |
| 24_1 | 22 | 2872442 | 2822493 |
| 24_2 | 22 | 3238322 | 3181139 |
| O_52 | 23 | 4824028 | 4736536 |
| O_33 | 24 | 8097430 | 7823335 |
| O_53 | 24 | 6950604 | 6823931 |
| O_54 | 24 | 7328098 | 7206891 |
| 7_6 | 25 | 4724222 | 4644675 |
| 7_8 | 25 | 3958230 | 3888586 |
| 7_9 | 25 | 5573382 | 5475996 |
| O_35 | 26 | 6353520 | 6200094 |
| 1_3 | 27 | 5437764 | 5335724 |
| 1_6 | 27 | 13460400 | 13226617 |
| 1_5 | 27 | 2693392 | 2643106 |
| 1_9 | 27 | 4532946 | 4463302 |
| O_32 | 28 | 7196432 | 7045828 |
| O_40 | 29 | 4866672 | 4783596 |
| O_48 | 29 | 5202480 | 5126470 |
| O_39 | 30 | 5628158 | 5528866 |
| O_38 | 31 | 7413614 | 7298908 |
| O_49 | 31 | 7883554 | 7754338 |
| O_50 | 31 | 4741530 | 4545076 |
| O_45 | 33 | 6225906 | 6128238 |
| O_46 | 33 | 8377988 | 8212856 |
| O_16_2 | 34 | 5709914 | 5616112 |
| O_16_1 | 34 | 4766644 | 4699340 |
| O_17_2 | 35 | 3718504 | 3637065 |
| O_17_1 | 35 | 6451300 | 6337785 |
| O_18_2 | 36 | 4006110 | 3932177 |
| O_19_2 | 37 | 6492874 | 6376403 |
| O_19_1 | 37 | 2768510 | 2708426 |
| O_44 | 38 | 5615322 | 5451229 |
| O_12_2 | 39 | 942242 | 806402 |
| O_14_2 | 40 | 3417666 | 3329304 |
| O_14_1 | 40 | 3713426 | 3642157 |
| O_41 | 41 | 3371914 | 3287796 |
| O_8_2 | 42 | 3584514 | 3502372 |
| O_8_1 | 42 | 3559174 | 3501733 |
| O_43 | 43 | 5575122 | 5476620 |
| O_2_1 | 44 | 3253714 | 3199028 |
| O_6_1 | 45 | 1662576 | 1623083 |
| O_6_2 | 45 | 3950142 | 3874702 |
| O_5_1 | 46 | 9185908 | 8971133 |
| O_5_2 | 46 | 4270268 | 4191280 |
| O_7_1 | 47 | 5570474 | 5479218 |
| O_3_2 | 48 | 4363274 | 4272662 |
| O_3_1 | 48 | 1277414 | 1012594 |
| O_10_1 | 49 | 1672408 | 1629728 |
| O_42 | 50 | 4539994 | 4446785 |
| O_13_1 | 51 | 2810912 | 2768817 |
| O_13_2 | 51 | 12861006 | 12617850 |

Table S2. Pairwise comparisons of taxon-specific SNPs in taxonomic groups ‘*Carex dacica*’, *C*. *bigelowii* ‘subsp. *bigelowii*’, *C*. *nigra*, and interpreted hybrids (see Table 1). N = number of individuals, amount of fixed alleles (AA/aa or aa/AA), private alleles occurring as heterozygotes in group 1 (Aa/aa) group 2 (aa/Aa), total amount of private alleles, and mdf (mean allele frequency difference between loci).

| Group 1 | Group 2 | N1 | N2 | fixed private alleles | private  alleles 1 | private  alleles 2 | total private alleles | mdf |
| --- | --- | --- | --- | --- | --- | --- | --- | --- |
| ‘*C*. *dacica*’ | ‘subsp. *bigelowii*’ | 77 | 27 | 132 | 7877 | 3898 | 11775 | 0.232 |
| ‘*C*. *dacica*’ | hybrids | 77 | 25 | 0 | 2604 | 3621 | 6225 | 0.110 |
| ‘*C*. *dacica*’ | *C*. *nigra* | 77 | 4 | 45 | 6599 | 761 | 7360 | 0.175 |
| ‘subsp. *bigelowii*’ | hybrids | 27 | 25 | 0 | 926 | 5922 | 6848 | 0.139 |
| ‘subsp. *bigelowii*’ | *C*. *nigra* | 27 | 4 | 410 | 5344 | 3460 | 8804 | 0.235 |
| hybrids | *C*. *nigra* | 25 | 4 | 36 | 8016 | 1212 | 9228 | 0.189 |
